# Supplementary material for: Nucleoplasmic signals promote directed transmembrane protein import simultaneously via multiple channels of nuclear pores
Source: Nat Commun. 2020 May 4;11:2184. doi: 10.1038/s41467-020-16033-x (PMC7198523; doi:10.1038/s41467-020-16033-x)
Supplement: Supplementary file 1 — Supplementary Information [file 41467_2020_16033_MOESM1_ESM.pdf]

Supplementary Information for

Nucleoplasmic signals promote directed transmembrane protein import simultaneously via multiple channels of nuclear pores

Authors: Krishna C. Mudumbi[1,2,3*], Rafal Czapiewski[4], Andrew Ruba[1], Samuel L. Junod[1], Yichen Li[1], Wangxi Luo[1], Christina Ngo[1], Valentina Ospina[1], Eric C. Schirmer[4*], Weidong Yang[1*]

Affiliations:

[1]Department of Biology, Temple University, Philadelphia, Pennsylvania 19122, USA.

[2]Department of Pharmacology, Yale University School of Medicine, New Haven, Connecticut 06520, USA.

[3]Yale Cancer Biology Institute, Yale University, West Haven, Connecticut 06516

[4]The Institute of Cell Biology, University of Edinburgh, Edinburgh EH9 3BF, UK.

***Correspondence to:** Krishna C. Mudumbi (krishna.mudumbi@yale.edu), Eric C. Schirmer (e.schirmer@ed.ac.uk) and Weidong Yang (weidong.yang@temple.edu)

INM Protein	N terminal domain (amino acids/kDa)	C terminal domain (amino acids/kDa)	NLS	ID Linker
**NET51**	4/0.5	10 /1.2	-	-
**NET59**	11/1.3	20/2.3	-	-
**NET99**	19/2.3	227/27	-	-
**LBR**	208/25	39/4.7	+	+
**LBR Δ63-172**	98/11.8	39/4.7	-	-
**LBR R74T**	208/25	39/4.7	-	+
**LBR ΔLinker**	166/13.9	39/4.7	+	-
**Lap2β**	410/45	24/2.9	+	+
**Lap2β-tNLS**	410/45	24/2.9	-	+

Supplementary Table 1. List of INM proteins used and their unique features. Each INM protein used in this work is listed in the left column. The next two columns give a description of the number of amino acids and the approximate molecular weight of each terminus (N or C). The following two columns show whether the protein has an NLS or ID domain (+) or not (-).

Protein	s (nm)	N	Radial Peak (nm)	σ_{TR} (nm)	Reproducibility	Points for ~90% Reproducibility
NET51	6.84	1037	41	1.14	99.97	150
NET59	6.62	762	40	1.26	99.46	150
NET99	6.46	1121	42	1.00	97.48	150
LBR-N	5.77	908	25	0.94	99.93	140
LBR-C	7.4	1058	41	1.26	99.91	150
LAP2β	6.08	1181	24	0.90	99.90	140
LBR Δ63-172	6.71	1059	39	1.09	99.89	150
LBR R74T	6.94	1132	40	1.11	99.94	150
LBR ΔLinker	6.19	940	39	1.03	99.77	150
LAP2β ΔNLS	6.22	638	41	1.26	99.24	150
LBR-N + WGA	6.9	729	43	1.37	95.62	150
LBR-N + anti-gp210	6.44	560	42	1.42	97.91	150

Supplementary Table 2. Transport route precision (σ_{TR}) for each INM protein candidate. Using the simulations we estimated the reproducibility of the final 3D transport routes. Where σ_{TR} is the transport route precision, *s* is the standard deviation of the 3D peak fitting, and *N* is the number of experimental single-molecule localizations collected and used to generate the 3D density map**.**

Protein	Radial Peak (nm)			Standard Error	Radial Peak (nm)
	-20 nm to 20 nm	-15 nm to 15 nm	-10 nm to 10 nm		
NET51	41	41	42	0.11	41
NET59	40	40	39	0.16	40
NET99	42	42	42	0.02	42
LBR-N	25	25	25	0.09	25
LBR-C	41	41	42	0.25	41
Lap2β-N	24	25	24	0.13	24
LBR Δ63-172	39	39	38	0.23	39
LBR R74T	40	40	39	0.15	40
LBR ΔLinker	39	39	38	0.36	39
Lap2β-C	68	68	68	0.09	68
Lap2β ΔNLS	41	40	41	0.22	41
LBR-N + WGA	43	43	43	0.10	43
LBR-N + anti-gp210	42	42	42	0.06	42

Supplementary Table 3. Curvature of the NE does not affect the determination of the correct transport route. The radial peak for different axial segments of proteins compared to the radial peak as found in the complete radial transformation. The standard error measures the error between the different transport models.

INM Proteins	NLS	≥65 AA ID linker	NLS+ID linker arranged appropriately	CF-GOR disorder prediction	All criteria met
NET97	x	✓	✓	x	x
NET38	x	x	x	x	x
NET55	x	x	x	x	x
NET51	x	x	x	x	x
NKP9	x	x	x	x	x
C2xorf3	x	x	x	x	x
NET50	x	x	x	x	x
Emerin	✓	✓	✓	✓	✓
NET20	x	x	x	x	x
NET37	x	x	x	x	x
mNET8	✓	x	x	x	x
LBR	✓	✓	✓	✓	✓
NET25	x	✓	✓	x	x
Man1	x	✓	✓	x	x
mNET20	x	x	x	x	x
NKP2	x	x	x	x	x
NET30	x	x	x	x	x
NET59	x	x	x	x	x
Nurim	x	x	x	x	x
mNET3	x	✓	✓	x	x
NET39	x	✓	✓	x	x
NET33	x	✓	✓	x	x
NKP13	x	x	x	x	x
NET46	x	x	x	x	x
NET34	x	✓	x	x	x
NET99	x	x	x	x	x
SUN1	x	✓	x	x	x
SUN2	✓	✓	x	✓	x
NKP39	x	✓	x	x	x
NET47	x	x	x	x	x
NET95	x	x	x	x	x
NET29	x	x	x	x	x
NET23	x	x	x	x	x
TMEM194	x	x	x	x	x
NET5	x	✓	✓	x	x
TMEM214	✓	✓	✓	x	x
mNET1	✓	✓	x	✓	x
NKP91	x	x	x	x	x
mNET11	x	x	x	x	x
LAP2	✓	✓	✓	✓	✓
LAP1	✓	✓	✓	✓	✓
NET9	x	✓	✓	x	x
IAG2	x	x	x	x	x
NKP68	x	✓	✓	x	x
C17ORF31	x	x	x	x	x
NKP83	x	x	x	x	x

ONM Protein	NLS	≥65 AA ID linker	NLS+ID linker arranged appropriately	CF-GOR disorder prediction	All criteria met
NKP66	x	x	x	x	x
CKAP4	x	✓	x	✓	x
mNET22	x	✓	x	✓	x
NKP64	✓	✓	x	✓	x
NET4	x	x	x	x	x
NET24	x	x	x	x	x
NET31	x	✓	x	x	x
NKP16	x	x	x	x	x
AYTL1	x	x	x	x	x
LRRC8A	x	✓	x	✓	x
FAM134A	✓	✓	x	✓	x

Supplementary Table 4. INM and ONM NETs used in bioinformatic analysis of NLS-dependent transport. 46 different ONM NETs and 11 different INM nets were analyzed for strong NLSs and long ID regions which are required to facilitate NLS-dependent transport into the INM. Details on the bioinformatic analysis is included in the Methods section (Bioinformatic analysis).

Protein	Mobile (A)	Immobile (1-A)	$\tau_{1/2}$ (s)
**wt LBR**	40±6%	60±6%	45.66
**LBR-tNLS**	41±5%	59±5%	78.5
**LBRΔLinker**	70±4%	30±4%	114.57

Supplementary Table 5. Mobile fraction and recovery rate as determined by ensemble FRAP experiments. Both the mobile and immobile fraction for LBR and its variants were determined from ensemble FRAP experiments. $\tau_{1/2}$ represents the time in seconds at which half the fluorescence was recovered after photobleaching.

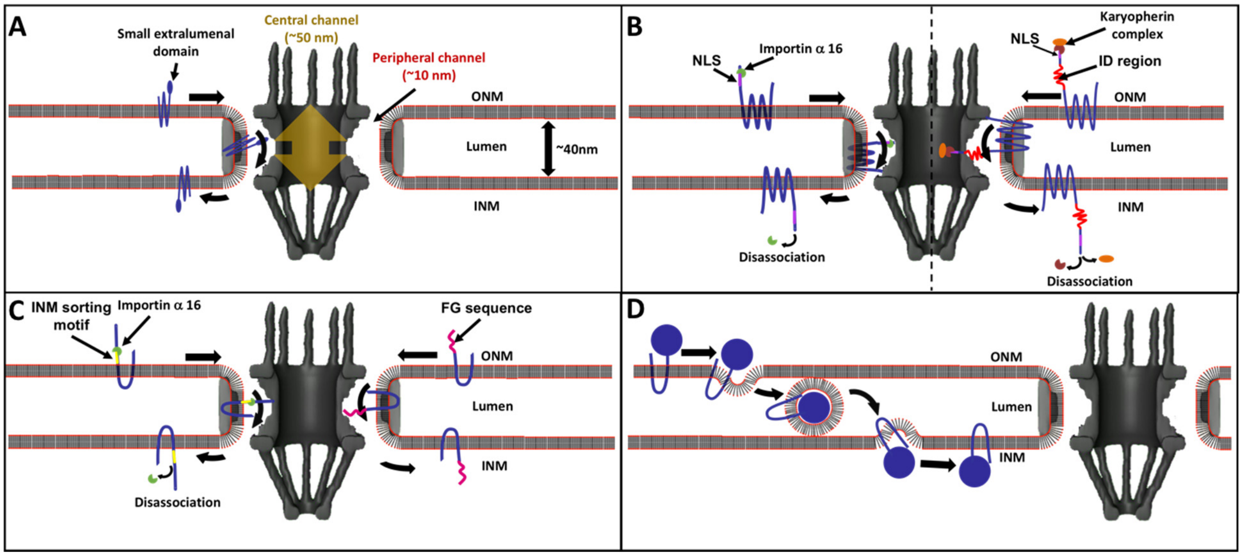

Supplementary Figure 1. INM protein transport models. (A) Lateral diffusion-retention. INM transmembrane proteins can passively diffuse through the peripheral channel of NPCs along the nuclear envelope without the help of transport receptors. Once the INM proteins diffuse from the ONM to reach the INM they are generally retained there by interacting with lamins, chromatin, or other nuclear proteins. (B) NLS-dependent facilitated transport. The transport of INM proteins might also occur along the membrane through the peripheral channels, similar to lateral diffusion, with the help of transport receptors. These INM proteins might contain a nuclear localization signal (NLS), which is recognized by transport receptors. They may then transport into the INM by going through the peripheral channel, though that may only be possible if the transport receptor is a short isoform of importin-α called importin-α 16 which is small enough to fit through the small peripheral channels. Alternatively, the NLS may be separated from the transmembrane domain by an intrinsically disordered (ID) domain. The NLS and transport receptor proteins might pass through the NPC central channel with the help of phenylalanine-glycine nucleoporins (FG Nups) while the ID domain passes through the scaffold of the NPC, which may be possible due to the high structural plasticity within the NPC. (C) In non-NLS signal-sequence mediated transport, an INM sorting motif might be recognized by importin-α 16 and then translocated through the peripheral channel of the NPC. Alternatively, this process might also be mediated by phenylalanine-glycine (FG) repeats on INM proteins which may then interact with FG-Nups found in the peripheral channel and facilitate transport. (D) Vesicle mediated transport avoids the NPC altogether by forming vesicles on the ONM carrying the INM protein through the lumen of the nuclear envelope where it next fuses with the INM, and there deposits the INM protein.

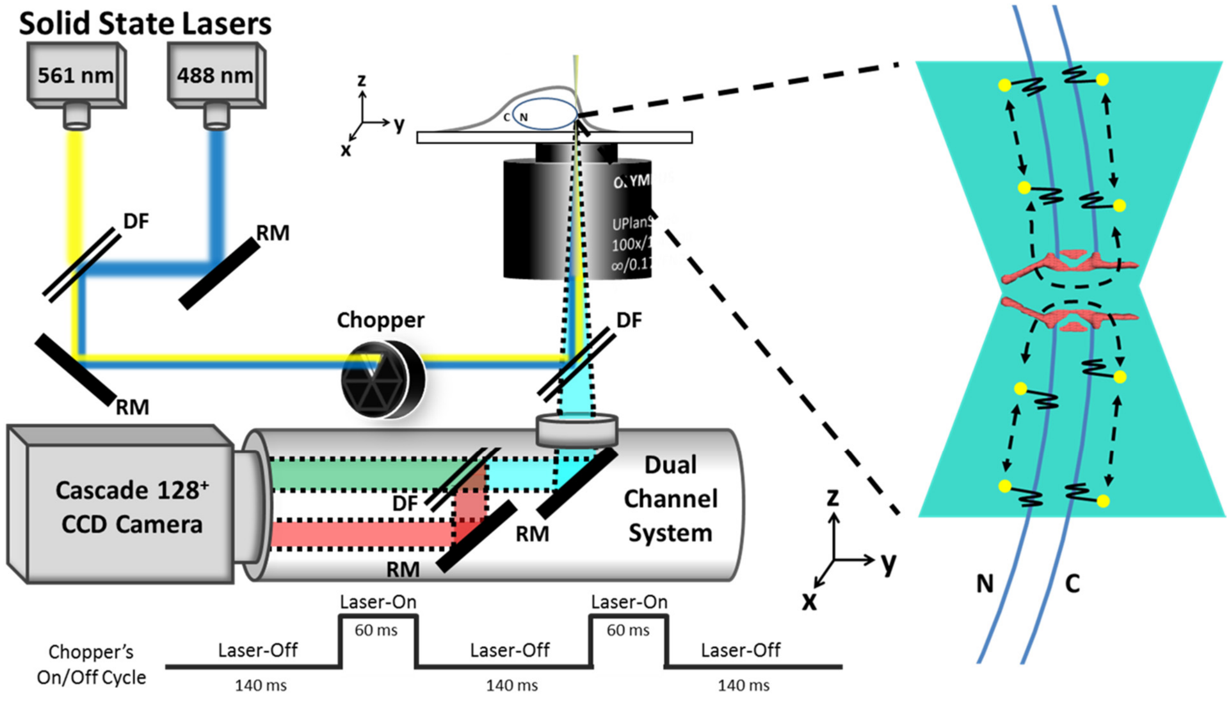

Supplementary Figure 2. Single-point edge-excitation sub-diffraction (SPEED) microscopy setup with a vertical illumination point-spread-function. Co-alignment of 488 nm and 561 nm solid-state laser lines was used to image GFP and mCherry, respectively. After passing through a mix of dichroic filters (**DF**) and reflection mirrors (**RM**), the light passes through a 100X Olympus objective and illuminates a single pore. The nuclear envelope (blue oval) is targeted at the equator of the cell with a single-point illumination laser (light blue hourglass shape, ~210 nm in the x and y dimensions and ~540 nm in the z dimension), and a single fluorescently tagged NPC is localized. Fluorescently tagged transmembrane proteins are subsequently tracked as they transit through the area. The reflected light (light blue light between dotted lines) will pass back through the objective and when applicable the light will be split into two channels by the dual channel system and then visualized by the Cascade 128^{+} CCD camera.

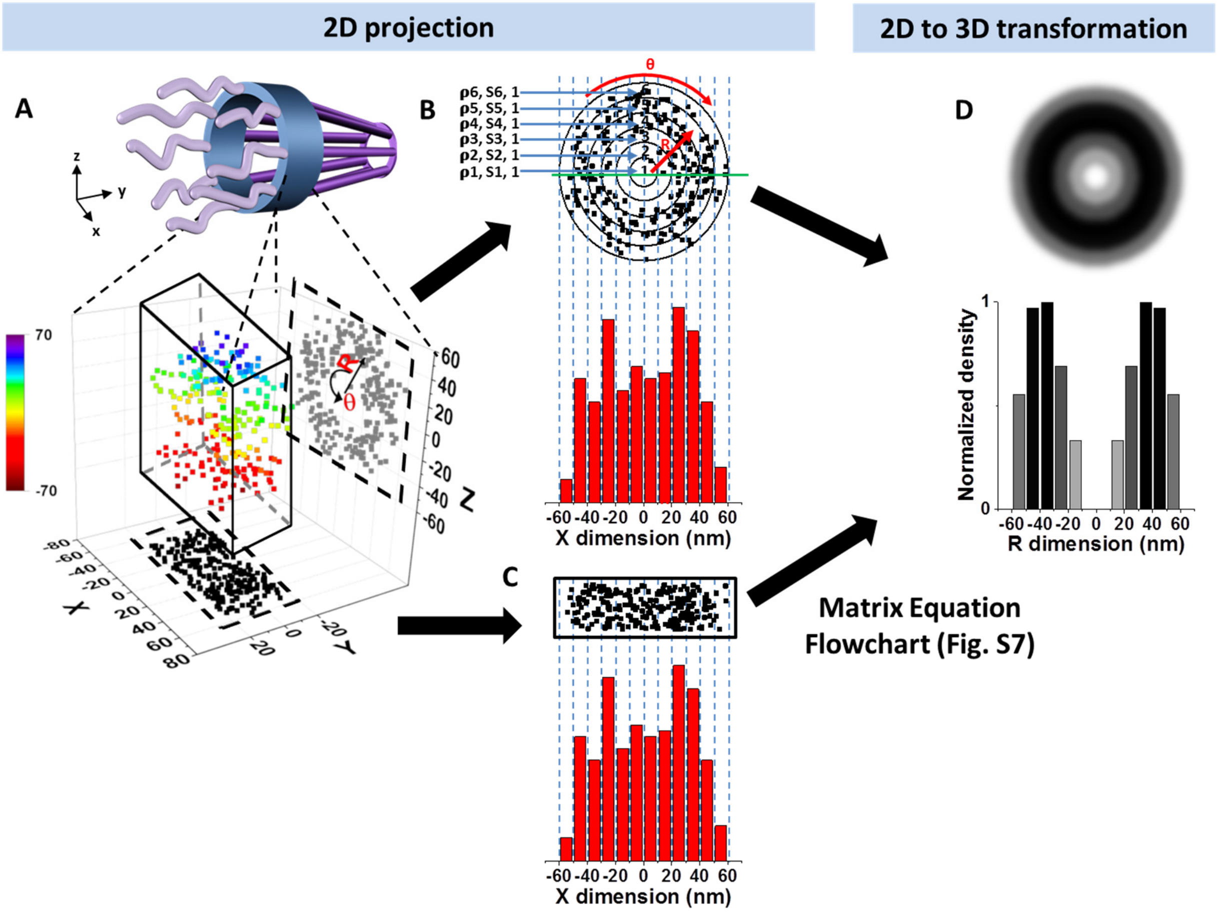

Supplementary Figure 3. A schematic demonstration, with simulated data, of the 2D to 3D transformation algorithms for molecules that diffuse through the NPC. (**A**) 3D spatial locations of randomly diffusing molecules inside the NPC can be coordinated in a cylindrical coordination system (R, Ɵ, Y) due to the cylindrical rotational symmetry of the NPC. The 3D molecular locations in the NPC (rainbow colored for Z position) are projected onto a 2D plane in a Cartesian coordination system (X and Y, shown as black points) by microscopy imaging (Y and Z shown as gray points). (**B**) The cross-sectional view of all the locations shown in Figure S5A (same as the gray points from the Y and Z dimension). These locations can be grouped into the sub-regions between concentric rings. Given the high number of randomly distributed molecules in the NPC the spatial density of locations ($\boldsymbol{\rho_i}$) in each sub-region ($\boldsymbol{s_{(i,j)}}$) between two neighboring rings will be rotationally symmetrical and uniform. These locations can be further projected into 1D along the Y dimension. The locations along the Y dimension can be clustered in a histogram with j columns. The total number of locations in each column ($\boldsymbol{A_{(i,j)}}$) is equal to $\mathbf{2} * \sum_{i=j}^{n} \boldsymbol{\rho_i} * \boldsymbol{s_{(i,j)}}$, which is further explained in Figure S7. (**C**) Histograms of 2D projected data from microscopy experiments are identical to B, thereby allowing us to use the aforementioned formula to determine the density of each concentric ring. (**D**) Using the algorithms, 2D projected data can be used to reconstruct the 3D spatial distribution of protein location on the NPC. Figures are reused with permission.

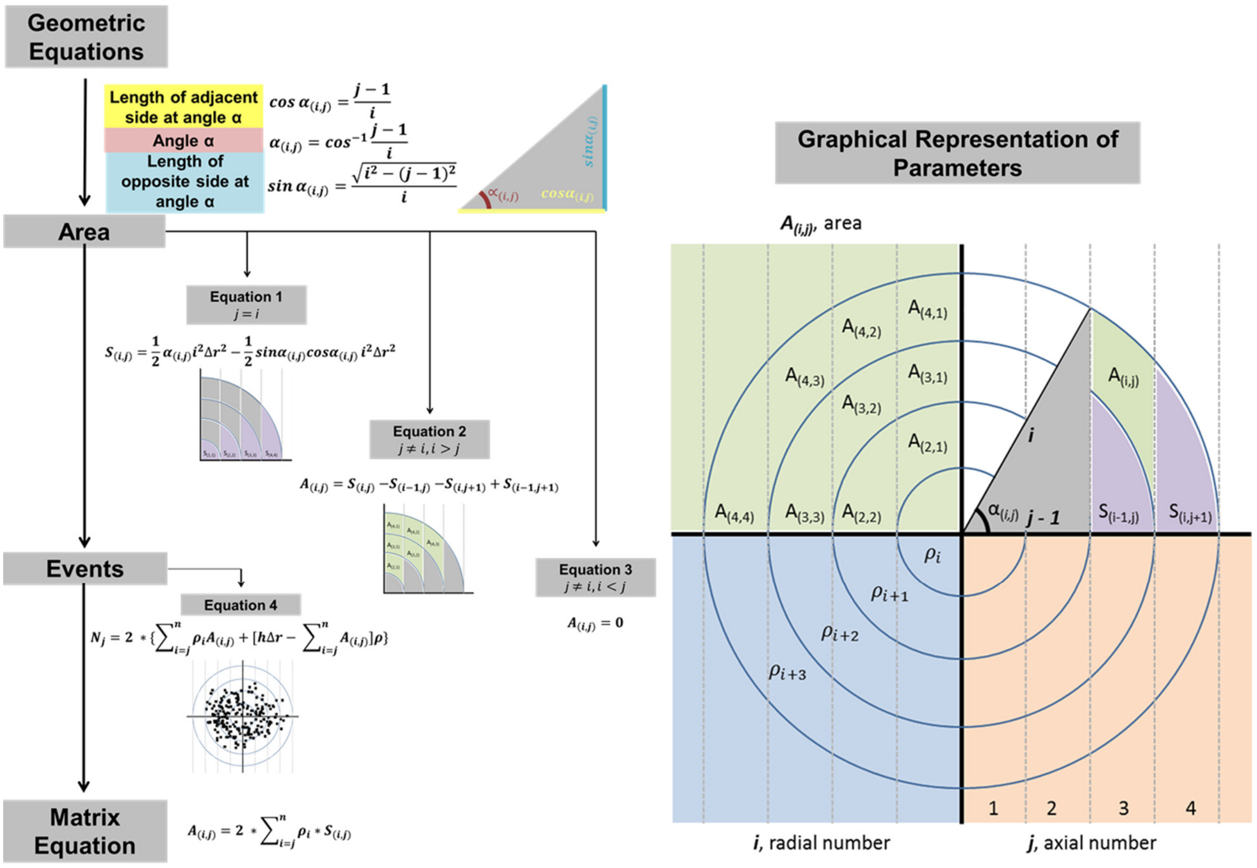

Supplementary Figure 4. Matrix equation flowchart. ($\boldsymbol{A}(\boldsymbol{i},\boldsymbol{j})$), area of the sub-region, (i), radial number, (j), axial number, ($\boldsymbol{\rho i}$), spatial probability density in each radial ring, ($\boldsymbol{S}(\boldsymbol{i},\boldsymbol{j})$), area of the sub-region, ($\boldsymbol{Nj}$), number of events, and ($\Delta\boldsymbol{r}$), bin size. Equations 1 – 3 will determine the area of the sub-region given certain parameters (i.e. i = j). Equation 4 will determine the number of events in the given area. Once the subregion area (($\boldsymbol{i},\boldsymbol{j}$)) and events ($\boldsymbol{Nj}$) are known, the spatial probability density in each radial ring ($\boldsymbol{\rho i}$) can be calculated. Figures are reused with permission.

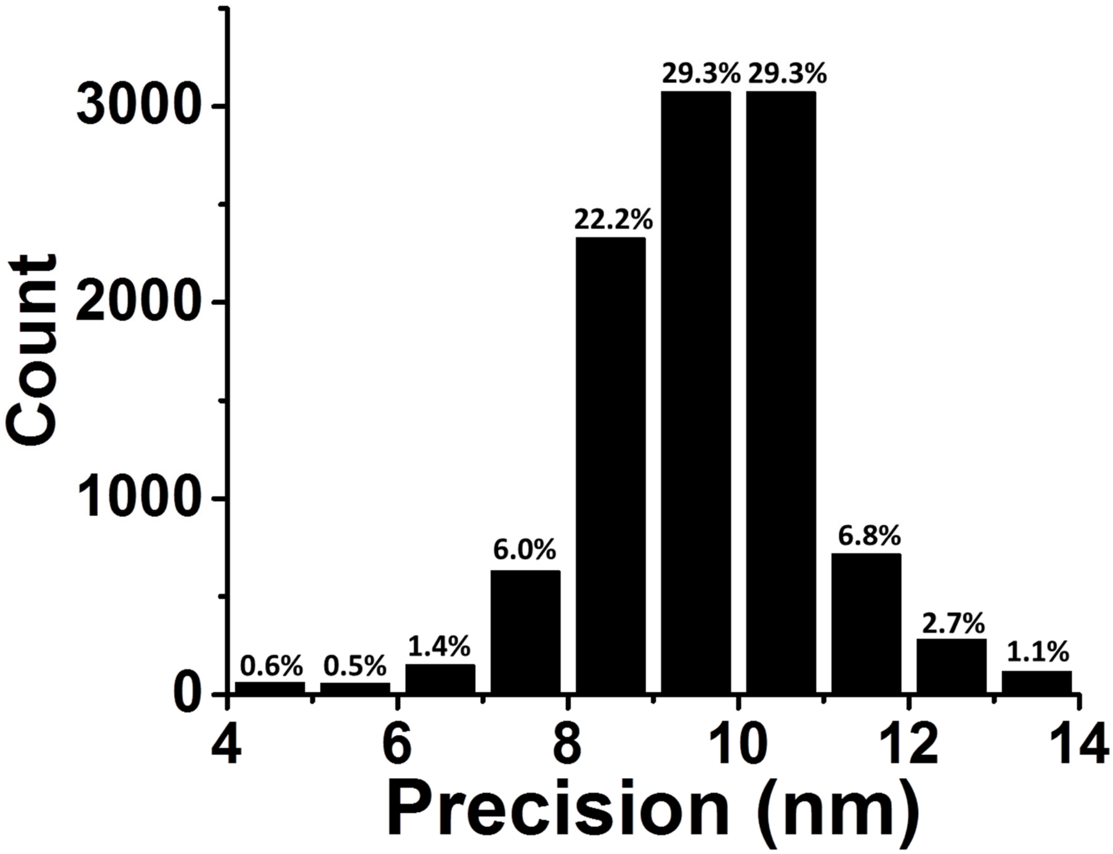

Supplementary Figure 5. Typical single-molecule localization precisions for INM proteins. On average, >2000 photons were collected from each single EGFP molecule at a frame rate of 0.4 ms. The average precision, based on all single-molecule localization events, is about 9.6±0.2 nm. Further details can be found in Methods.

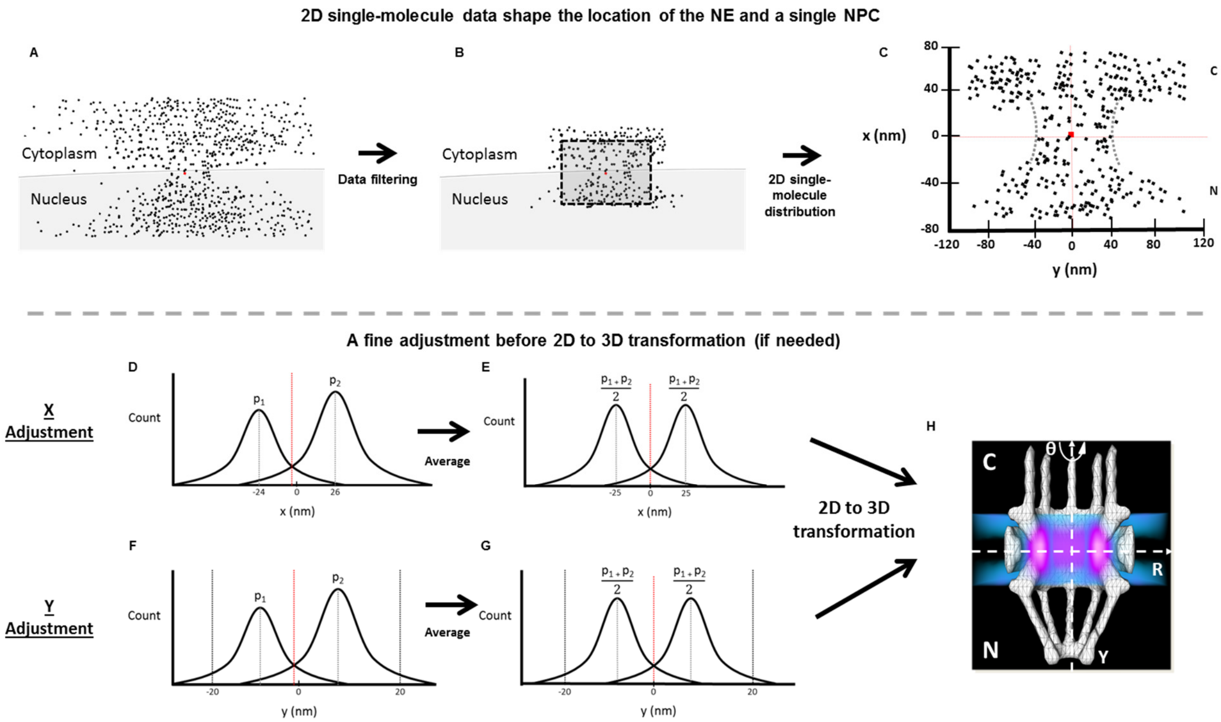

Supplementary Figure 6. Determination of the central axis of the NPC illuminated by SPEED microscopy.

Diagrams are used to demonstrate the detailed steps in our data analyses. (**A**) The initial single-molecule tracking data (black points) are plotted around the NPC's marker for the central position (red dot) collected with SPEED. (**B**) The collected data after filtering by using single-molecule localization filters (photon count, width of emission PSF). (**C**) The filtered 2D single-molecule data shape the spatial location of the NE and a single NPC. Furthermore, the 2D single-molecule distribution indicates the orientation of NPC, which must fall within a free angle of 1.4° perpendicular to the NE, for the data to be processed. The red dot represents the localized centroid of a fluorescently tagged NPC. Periodically, the localized NPC position does not perfectly overlap with the localized single-molecule data from the fluorescently tagged INM proteins and must be corrected. (**D-G**) Due to the rotational symmetry of the NPC, 2D single-molecule data is expected to be equally distributed along the central axis in the X and Y dimensions. Histograms are prepared for the X and Y dimensional data, and if the histograms are asymmetrical, as in this example, fine adjustments are made to restore symmetry using the formula $p_1 - \frac{p_1+p_2}{2}$. If the peaks are symmetrical, this step is skipped. (**H**) The 2D single-molecule data will then undergo the 2D to 3D transformation (**Fig. S6**) to produce the 3D density map. Figures are reused with permission.

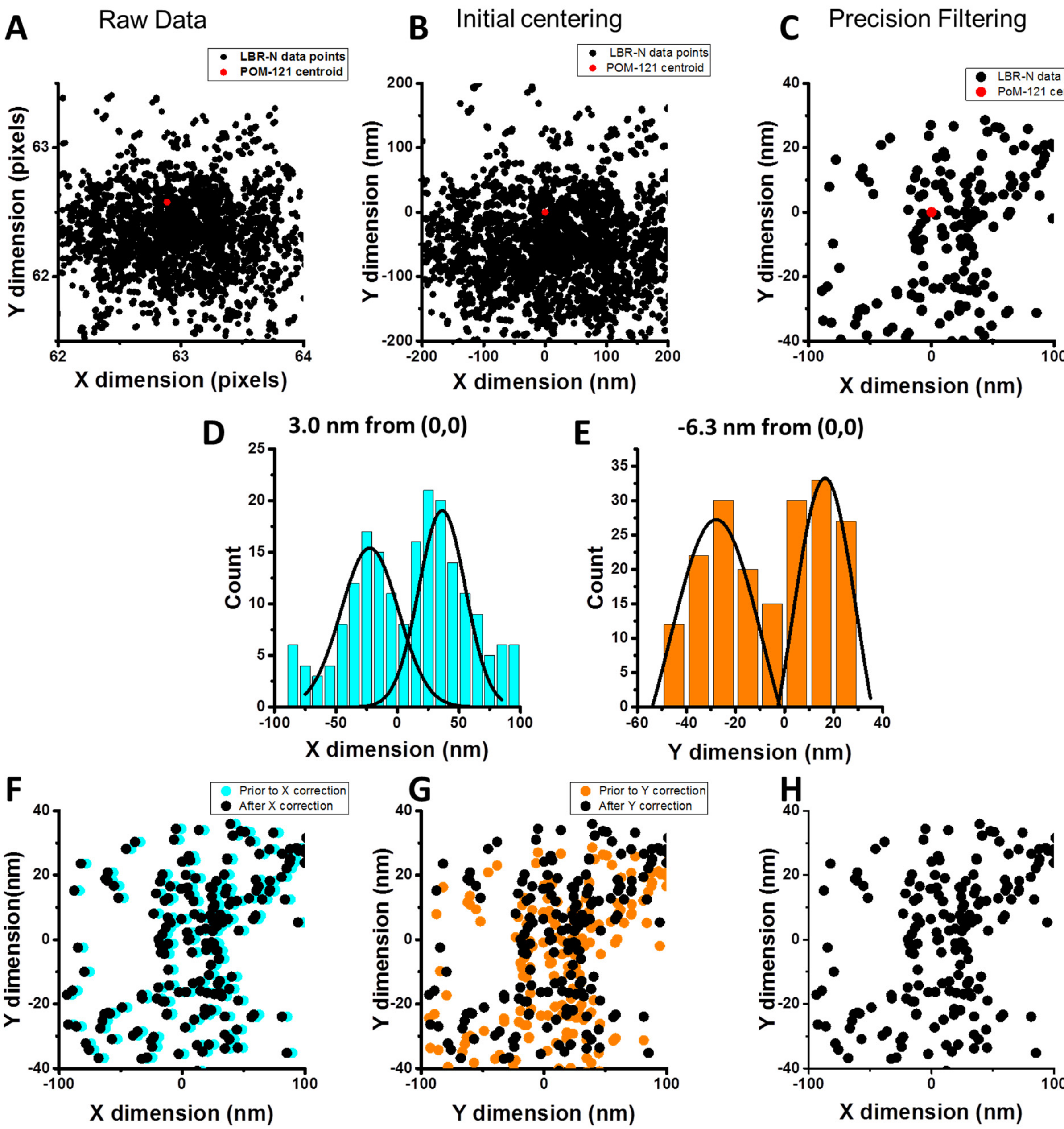

Supplementary Figure 7. An example of determining the centroid of NPCs with a real data set. (A) Single-molecule images of both an mCherry tagged NPC (red point) and EGFP tagged NETs (black points) are taken, localized using Gaussian fittings, and overlaid (dimensions shown in pixels). (B) The localized NPC is used to center the EGFP tagged NETs around the (0,0) position (dimensions shown in nm). (C) The raw data is then filtered and selected based on two single-molecule localization precision parameters (single-molecule photon count and width of the emission PSF, as detailed in the “Localization precision of isolated fluorescent spots in SPEED microscopy” of Methods), and overlaid on the originally localized NPC (red). (D, E) Next, X and Y histograms are created and fit with Gaussian functions. Since the NPC is considered to be cylindrically symmetrical, single-molecules in the X and Y dimension should be distributed about symmetrically. Therefore, the fittings from Gaussian functions can be used to center the data in the X and Y dimensions. (F) Original data is shown in light blue, and the corrected data

for the Y dimension based on fittings from D are shown in black. (G) Original data is shown in orange, and the corrected data for the X dimension based on fittings from E are shown in black. (H) Final data plotted after making the corrections for both the X and Y axis.

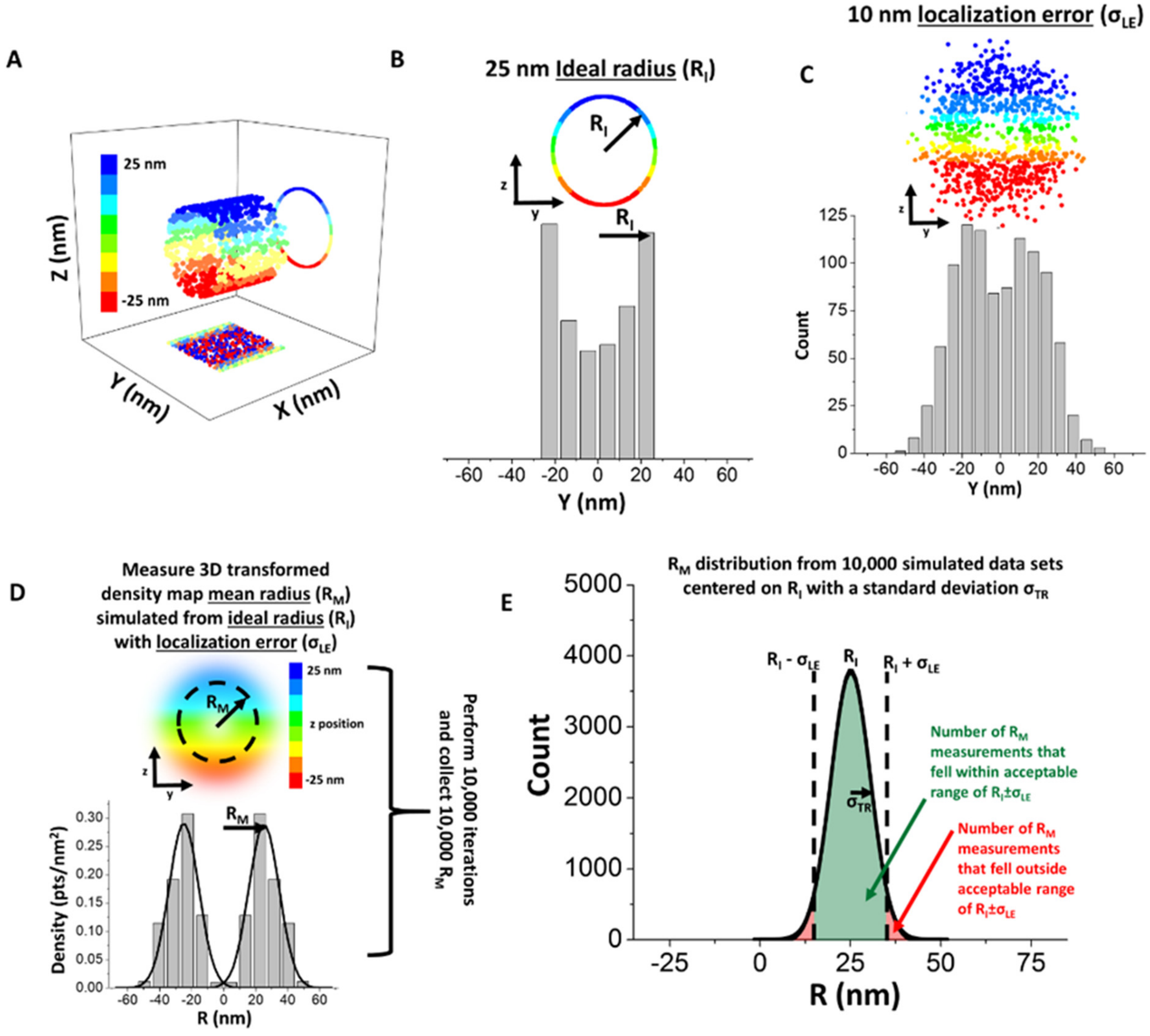

F

$$\textit{Reproducibility}\ \% = \frac{\#\ \textit{iterations within acceptable range}}{\textit{Total \# of iterations}} \times 100\%$$

Supplementary Figure 8. SPEED microscopy and 3D transformation reproducibility percentage using a simulation-based approach**.** For any given set of simulated data, the bin size is varied from 1 nm to the precision that is 10 nm in this example. **(A)** Data sets were simulated in three dimensions. Color bar indicates z position of the simulated points. **(B)** Each data set was simulated first with an ideal 25-nm radius (RI). (C) Subsequently, a localization error (σ_{LE}) of 10 nm was added to each point. **(C)** Using a 5-nm bin size for demonstration, the 2D histogram of the simulated data set with a 25-nm radius and 10-nm localization precision was determined. **(D)** 10,000 data sets were simulated with an ideal 25-nm radius (R_I) and a localization error (σ_{LE}) of 10 nm. The resultant 3D histograms were then each fitted with a Gaussian function to localize the mean position of each peak, which is designated as the mean radius R_M. **(E)** The histogram

for all the R_M values was determined and the number of simulated data sets that fell within the acceptable range of $R_I \pm \sigma_{LE}$ were counted. The acceptable range of $R_I \pm \sigma_{LE}$ was chosen because, in principle, the Rayleigh criterion limited the resolution of any single 3D histogram to the spread of that distribution, which was due to the simulated localization error (σ_{LE}). After 10,000 simulations, the histogram for R_M values converges on the mean (R_I) from which they were originally sampled, while the spread of the R_M histogram (σ_{TR}) converges on a value that is due to the number of simulated points in each distribution and the simulated localization error. **(F)** Reproducibility percentage was defined as the number of R_M values that fell within the acceptable range of $R_I \pm \sigma_{LE}$ divided by the total number of simulated data sets and multiplied by 100%.

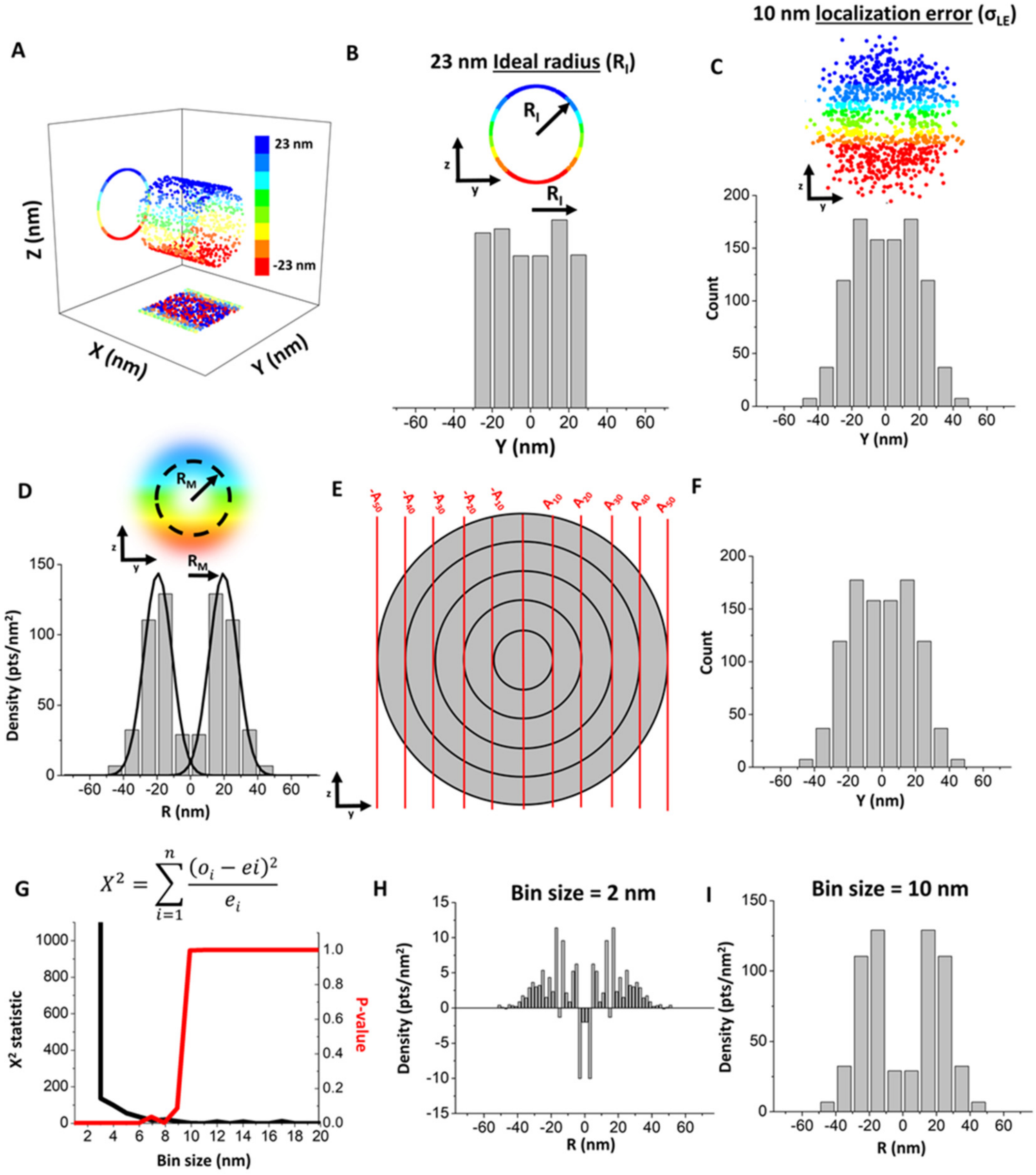

Supplementary Figure 9. Optimal bin-size determination using Chi-square error and P-value analysis. For any given set of simulated data, the bin size is varied from 1 nm to the precision that is 10 nm in this example. (**A**) Data sets were simulated in three dimensions. Color bar indicates z position of the simulated points. This representative data set contains 1,000 single molecule locations. (**B**) Each data set was simulated first with an ideal 23-nm radius (RI). (**C**) Subsequently, a localization error (σLE) of 10 nm was added to each point. Using a 10-nm bin size for demonstration, the 2D histogram of the simulated data set with a 23-nm radius and 10-nm localization precision was determined. (**D**) The 3D density histogram was then obtained via the 2D to 3D transformation algorithm and the peaks were fit with Gaussian distributions. (**E**) The 10-nm bin-size area matrix was calculated and multiplied by the 3D density distribution in (**D**) to reconstruct the 2D distribution (any negative values in the density distribution were set to zero) as shown in **F**. (**F**) The values of the reconstructed 2D distribution were then compared bin-by-bin to the original 2D distribution (as shown in **C**) using the Chi-square analysis equation

where ‘o’ refers to the observed histogram values in (**F**), ‘e’ refers to the expected histogram values in (**C**), ‘i’ refers to the bin, and ‘n’ refers to the total number of bins with histogram values in them. (**G**) The Chi-square test statistic and p-value were then plotted across the potential bin size values. A p-value ≤ 0.01 indicates that the 2D histograms in (**C**) and (**F**) are different from each other, suggesting the lack of enough data to allow sufficient sampling from each bin. On the other hand, a p-value > 0.99 suggests that the 2D histograms are statistically similar and likely have enough data points to accurately measure the value in each bin. Chi-square analysis was performed 1,000 times for each set of simulation parameters. (**H**) A 3D density histogram displaying large errors (negative density values) due to a bin size selection that was too small. Such errors occur when the single molecule density is not uniform throughout a given radial bin. This non-uniformity is due to under-sampling. (**I**) A 3D density histogram displaying no errors and a clear Gaussian distribution due to a properly selected bin size. A larger bin size can smooth the effects of under-sampling to a certain degree. Figures are reused with permission.

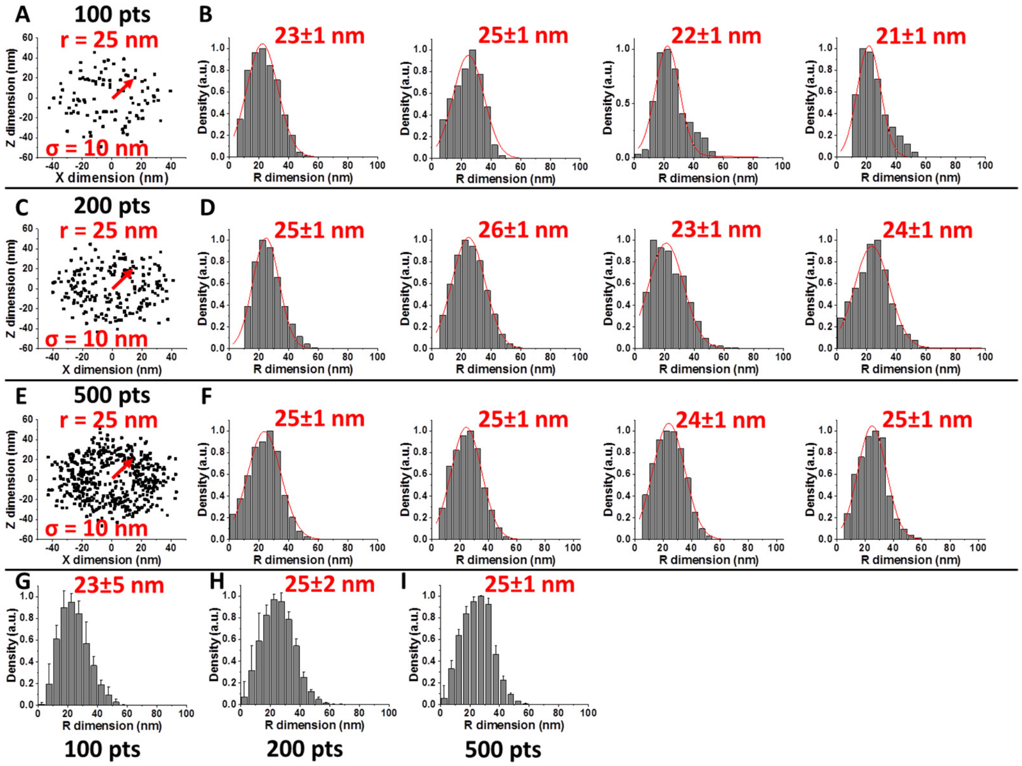

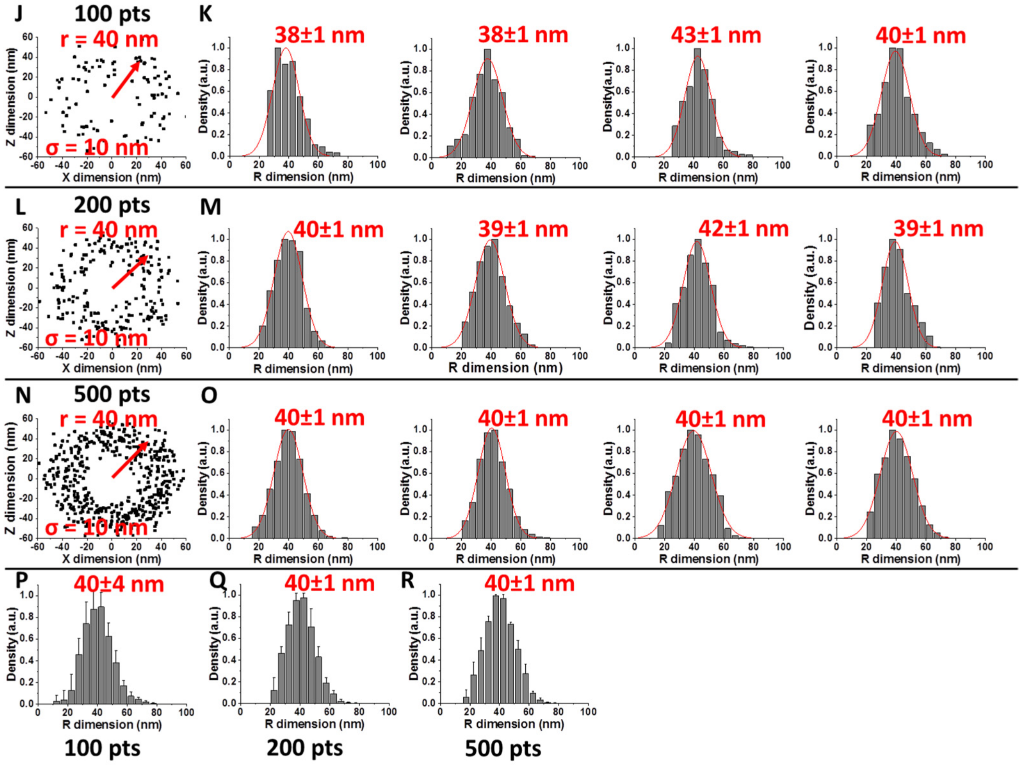

Supplementary Figure 10. Simulated data was used to estimate the minimum number of single-molecule localizations required to generate a reliable 3D probability density map with the

determined single-molecule localization precision. (A) 100 computationally generated single-molecule localizations randomly sampled from a normal distribution with a radius (r) of 25 nm (corresponding to LBR-N and Lap2β-N as determined in our experiments). A localization precision of 10 nm, similar to the experimental determined localization precision in this work, was simulated for each point by sampling from a normal distribution with $\sigma = 10$ nm. (B) Using the 2D to 3D transformation algorithms, Y-dimensionally projected data was transformed to 3D R-dimension density distributions for four different data sets (100 points each). The number above each figure indicates the fitted peak position ± the fitting error. (C-D) Simulation results based on 200 single-molecule localizations. (E-F) Simulation results based on 500 single-molecule localizations. (G-I) Average 3D density histograms from 100, 200, and 500 point simulations respectively (n=100). Error bars represent the variability in the histogram bin heights, while the number above the peak is the average peak position ± the route localization precision. This indicates that 500 points are sufficient to obtain a route localization precision under 1.5 nm. (J-R) Simulations and calculations were also performed, as described above, for simulated single-molecule localizations with a normal distribution and a radius of 40 nm (corresponding to INM proteins traveling through the peripheral channels of the NPC). As described above, error bars in (P-R) represent the variability in the histogram bin heights, and the number above the peak is the average peak position ± the fitting error. Figures are reused with permission.

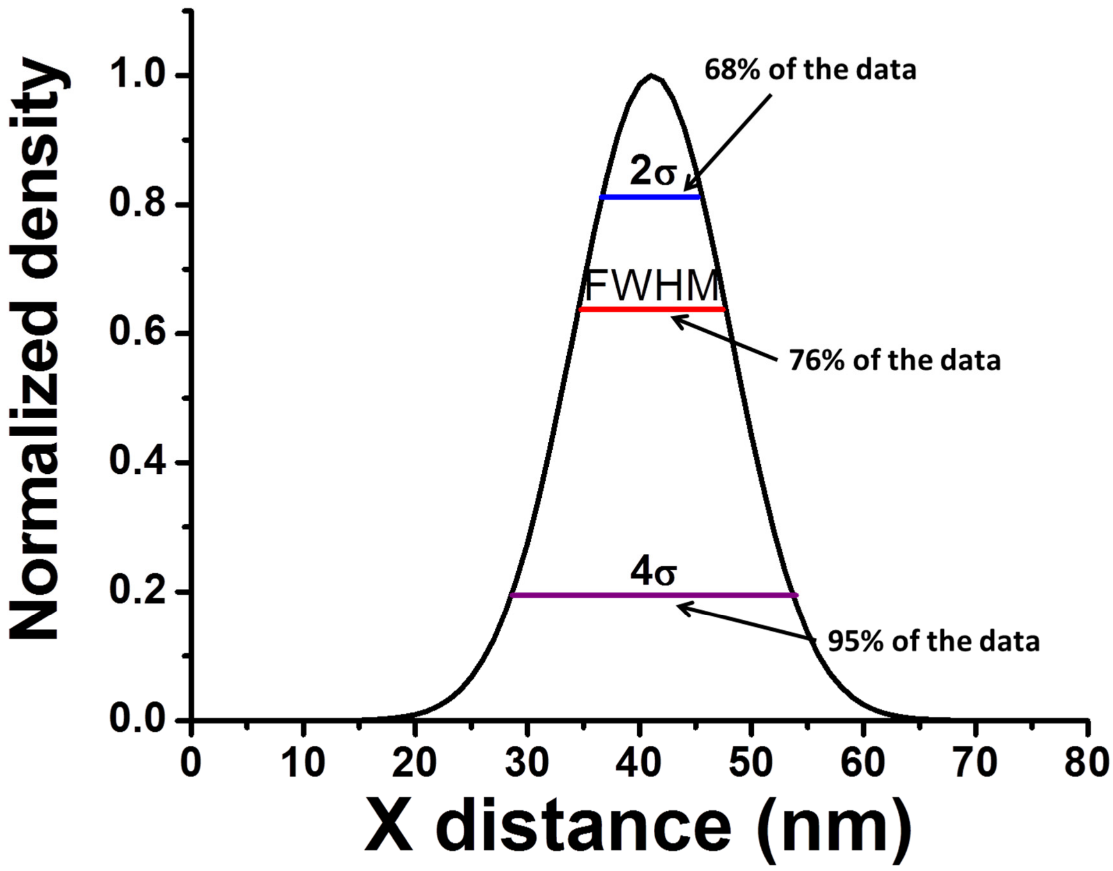

Supplementary Figure 11. Determination of route width by fitting the 3D spatial probability density distributions using a Gaussian function. Here we highlighted the full widths at one standard deviation (2σ), full width at half maximum (FWHM) and two standard deviations (4σ) with the corresponding probabilities of including single molecule locations in Gaussian fitting. Given the single-molecule localization error (σ_p) is not zero, in this study the following equation was used to find the corrected width of the transport route (W_c): $W_c = \sqrt{W_f^2 - \sigma_p^2}$, where W_f is the fitted width (obtained from either 2σ, FWHM, or 4σ), and σ_p is the single-molecule localization precision.

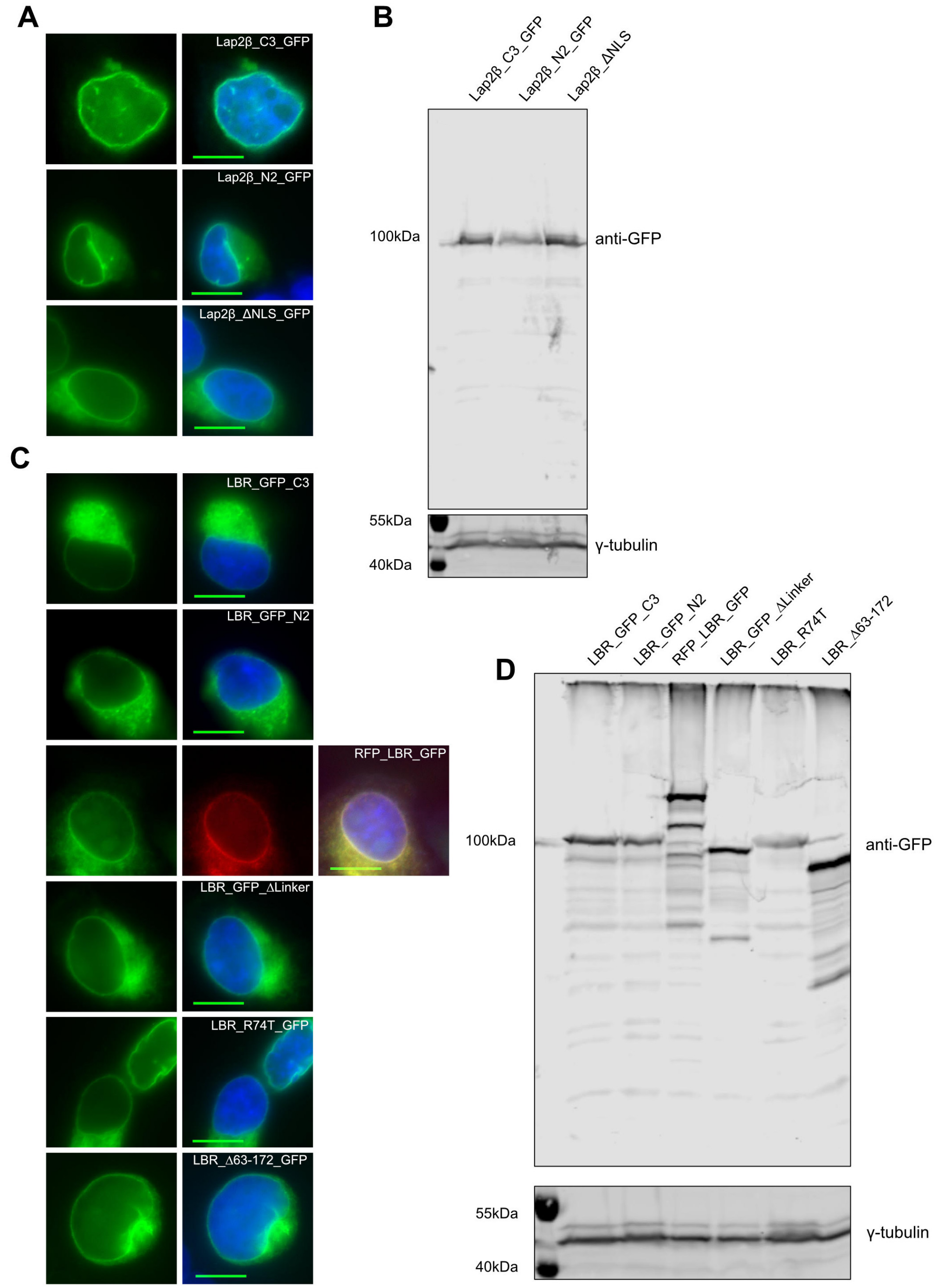

Supplementary Figure 12. Protein expression and localization for Lap2β and LBR constructs. The localization, membrane insertion, and expression for wild-type Lap2β and its mutated forms (A and B), as well as wild-type LBR and its mutated forms (C and D) used in this work. All scale bars at 5 µm, Western blots experiments were generated from at least 2 biological replicates, and fluorescence images are representative from at least 8 biological replicates.

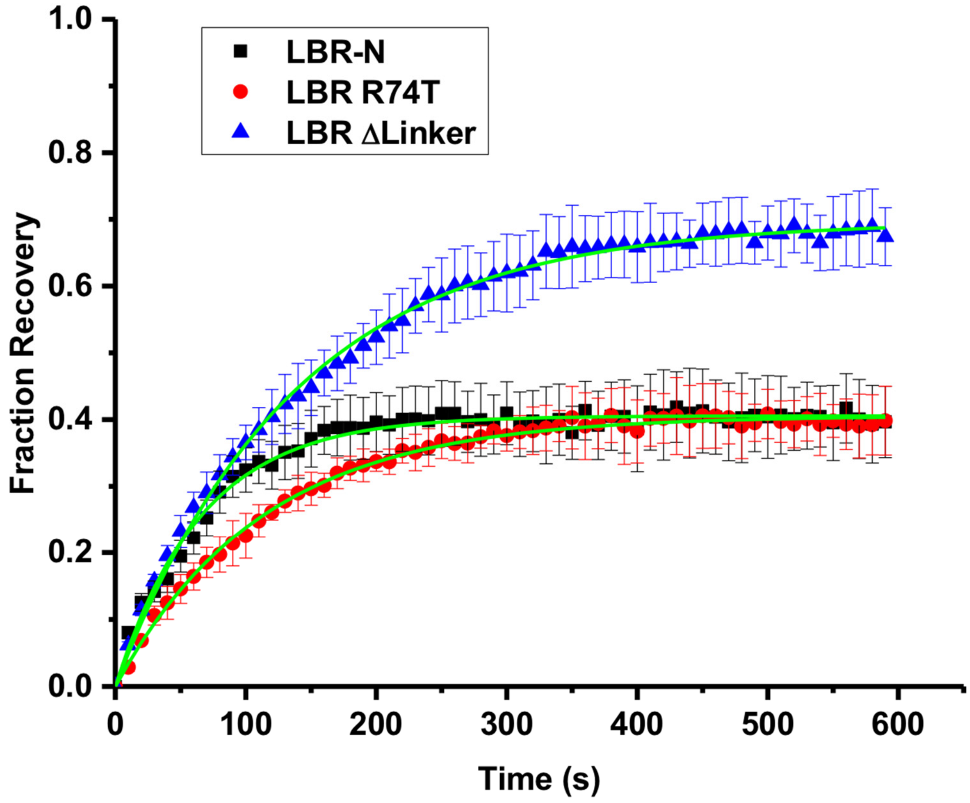

Supplementary Figure 13. FRAP curves for LBR, LBR R74T and LBR ΔLinker. Photobleaching was performed with an argon laser (488-nm laser line) for about 5 s to bleach an area of approximately 5 μm^2. Fluorescence recovery was measured every 5 s until the fluorescence reaches a plateau stage. Exponential functions have been applied to fit all measured curves averaged on six different nuclear envelopes (n = 6, error bars represent ± the SD). The following two formulae were used to calculate the immobilized fraction of INM proteins on the INM: $N_{ONM} + (1 - x) * N_{INM} = F_m(N_{ONM} + N_{INM})$ and $\frac{N_{ONM}}{(1-x)*N_{INM}} = R$. Here, the actual number of INM proteins on the ONM is N_{ONM} and on the INM is N_{INM}. For most INM proteins, previous measurements suggested that their ONM mobile fraction is almost 100% and here the INM immobile fraction is defined as x. F_m is the mobile fraction as determined by FRAP, and R is the corrected concentration ratio after considering the effect of the diffusion coefficient of the LBR protein in question (see Table 1).

Supplementary Note 1

A detailed post-localization 2D to 3D transformation process

Section Outline

I. Software Download ……………………………………………………………………………………………. pg. 1

II. Bin Optimization ………………………………………………………………………………………………. pg. 2

III. Axial to Radial Transformation……………………………………………………………………………….. pg. 4

IV. Simulation to Determine Confidence and Route Precision………………………………….……….. pg. 13

Section I
Software Download

Python 3

1) Install Python 3
   i. Since these scripts make use of several Python 3 libraries (tkinter, csv, random, os, sys, numpy, scipy, and math), the simplest way to install Python 3 with these required libraries is through the Anaconda distribution of Python 3 which can be found here (https://www.anaconda.com/download/).
   ii. Download the appropriate installer for your operating system (32-bit or 64-bit - Windows or Mac)
   iii. Once the installer has finished downloading, open it and follow the click-through instructions to install the Anaconda distribution of Python 3. An example of the first step of the installer for a 64-bit Windows system is shown below.

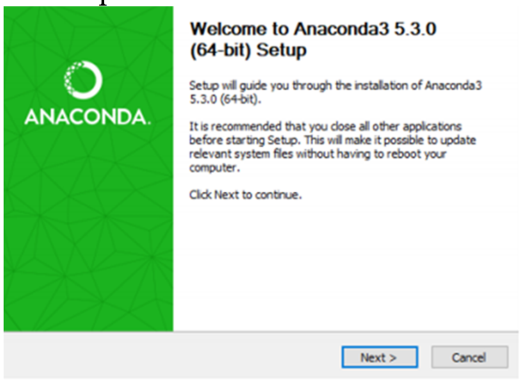

2) Download the script files

i. Follow the URL link below.
   i. https://github.com/SamJunod/yanglab
ii. Select the "Clone or Download" button and download the ZIP file. Extract the ZIP file to your preferred file location.

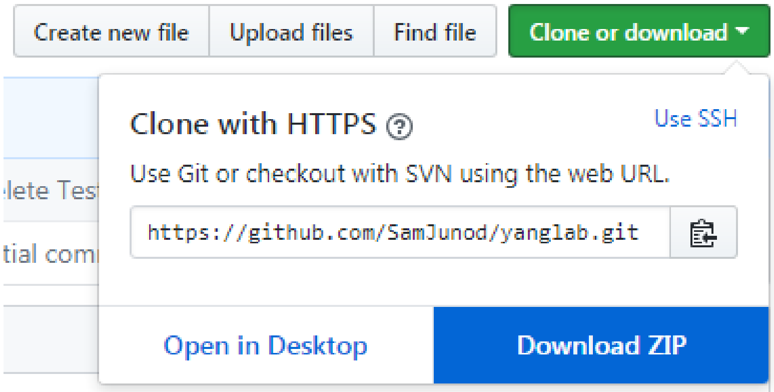

MatLab

1) Go to MatLab website, MATLAB - MathWorks - MATLAB & Simulink, and download the MatLab software.
   i. URL: https://www.mathworks.com/products/matlab.html

OriginLab

2) Go to OriginLab website and download the Origin software.
   i. URL: https://www.originlab.com/demodownload.aspx

Section II

Bin Optimization

1) Packaged in the Anaconda distribution of Python 3 is an integrated development environment for Python 3 called "Spyder." Open Spyder – it should have an icon on your desktop or an icon in the program list under the start menu for Windows.

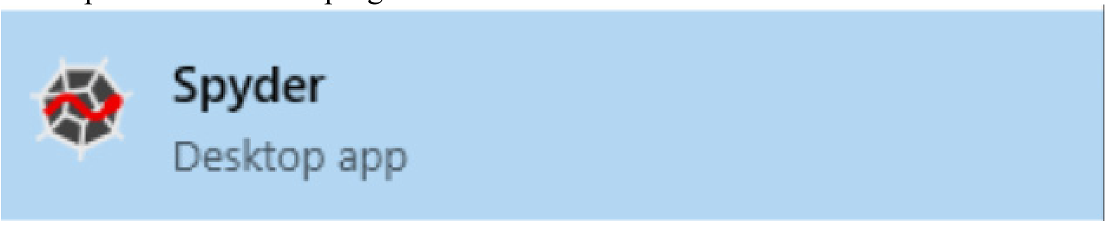

2) In Spyder, open "bin_size_optimization.py" using the File>Open menu.

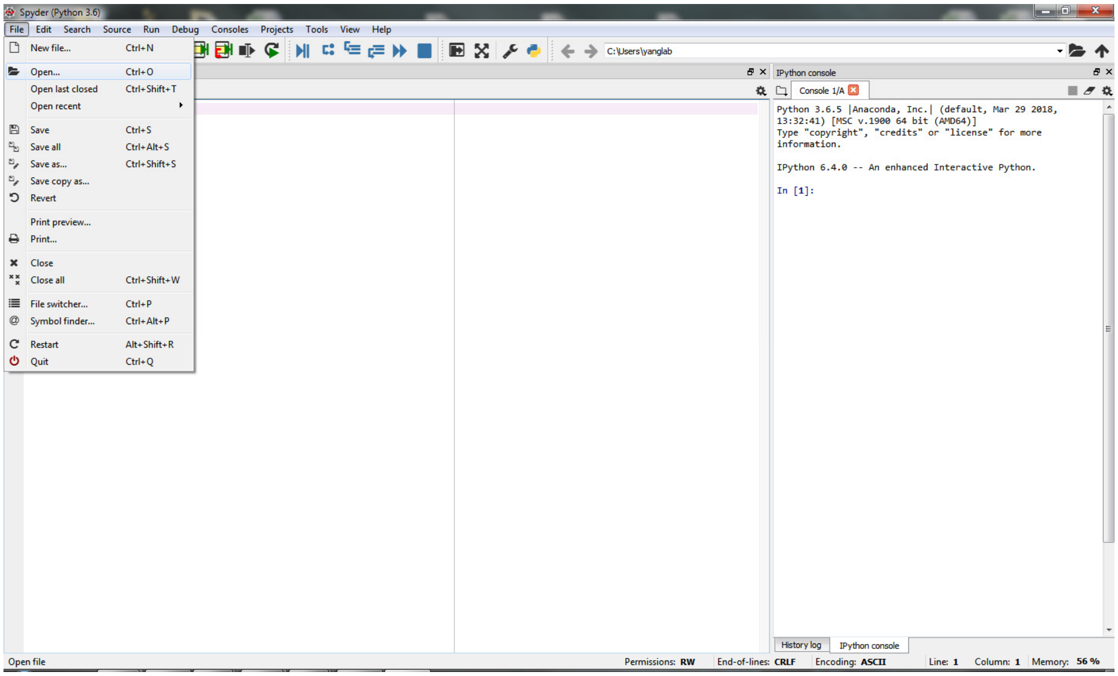

3) Then click the green “Run file” arrow in the toolbar.

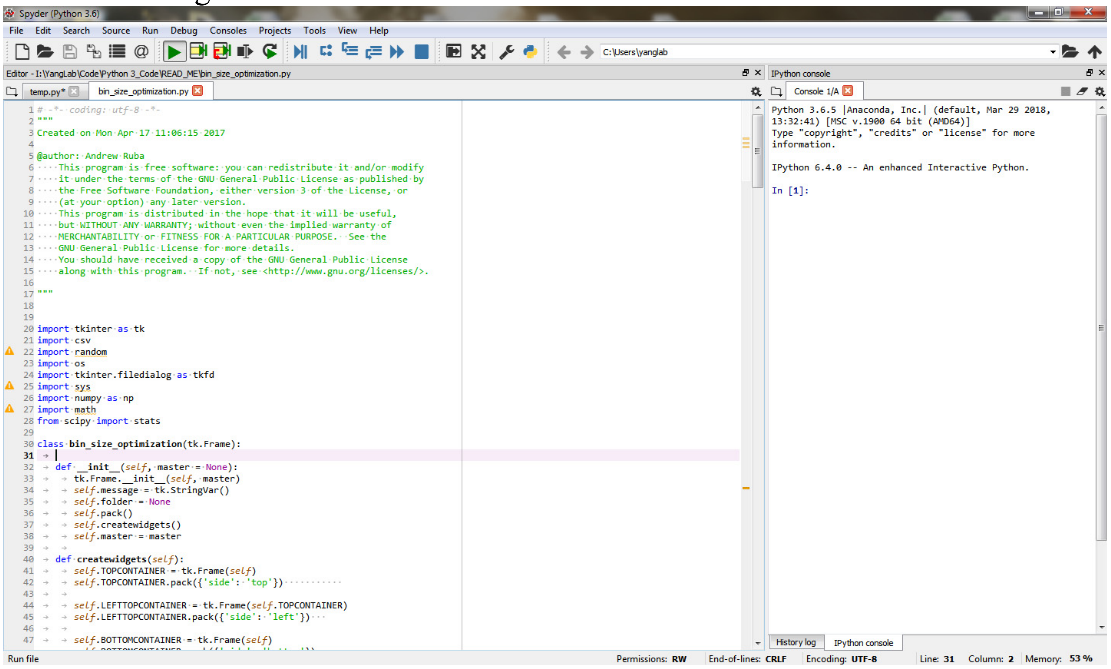

4) Once the script begins to run, a pop-up window will appear. Select the Optimize bin size button and then select your data in csv format.

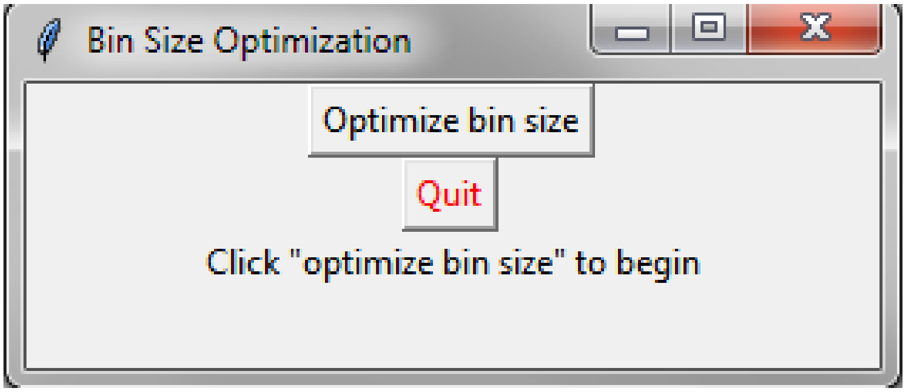

5) After the data is selected the script will run and output the ideal bin size. For LBR-C, the ideal bin size was determined to as 6nm.

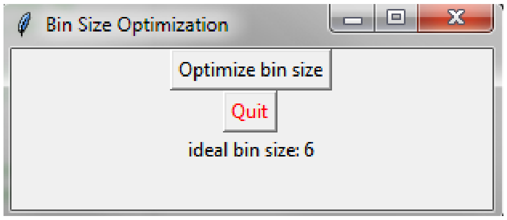

6) Select the “Quit” button to close the pop-up window.

Section III

Axial to Radial Transformation

1) Start-up MatLab and Open the “Axial_Radial_Transformation.m” file.

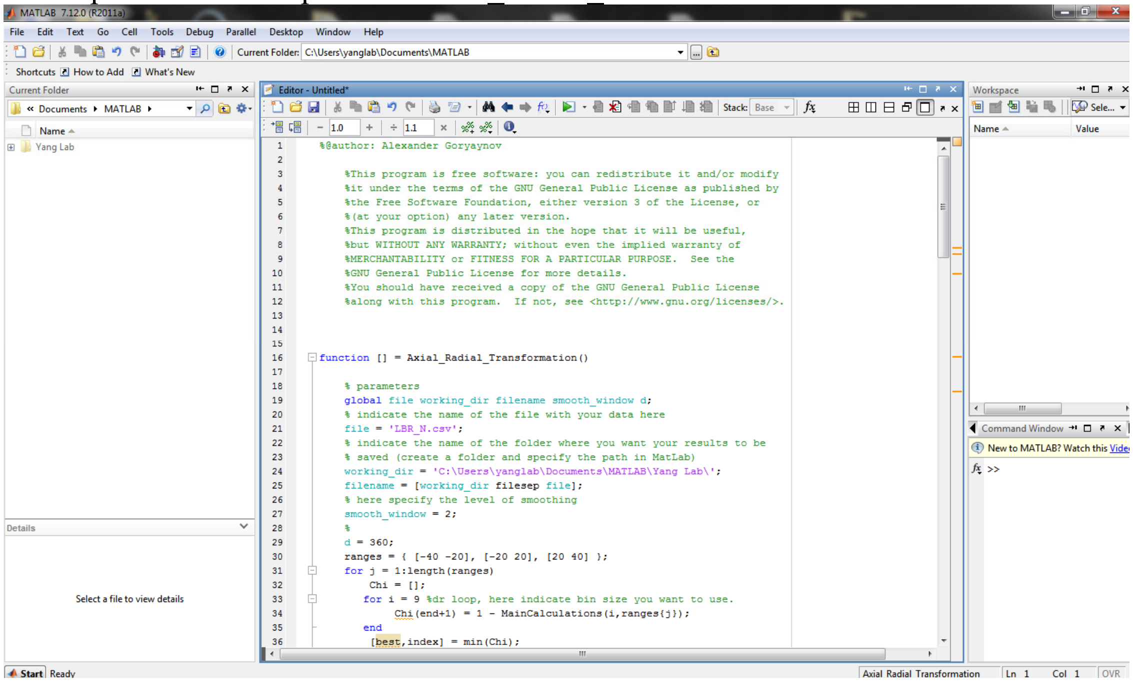

2) The MatLab script (Axial_Radial_Transformation.m) and the 2D localizations (LBR_C.csv) will have to be in the same folder.

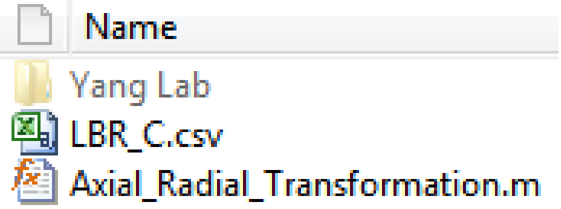

3) In the MatLab script, under the Editor box, make the following edits:

   i. File name (line 21, file = ‘***file name.csv***’;)

```
20      % indicate the name of the file with your data here
21      file = 'LBR_C.csv';
```

   ii. Working directory(line 24, working_dir = ‘***folder location of data***’;)

```
22      % indicate the name of the folder where you want your results to be
23      % saved (create a folder and specify the path in MatLab)
24 -    working_dir = 'C:\Users\yanglab\Documents\MATLAB\Yang Lab\';
```

   iii. Axial ranges (line 30, ranges = { [***axial ranges***] , [***for data***] };

```
30 -    ranges = { [-40 -20], [-20 20], [20 40] };
```

   iv. Binsize (line 33, for i = ***optimal bin size***). This is the bin size determined from section II.

```
33 -    for i = 6 %dr loop, here indicate bin size you want to use.
```

4) Select “Save” and run the script by selecting the green arrow in the toolbar under the “Editor” box.

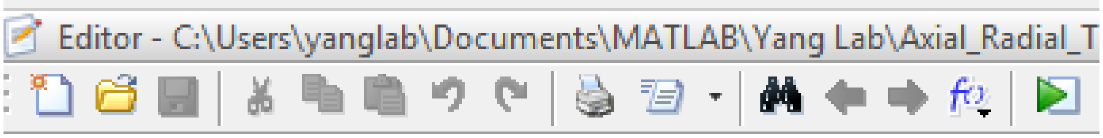

5) The program will write several items to the folder selected in the MatLab script.

Axial_Radial_Transformation.m
LBR_C.csv
R_96_dr_6_range_20-40.csv
R_96_dr_6_range_20-40_Chi_0.99764.txt
R_96_dr_6_range_20-40_m.png
R_96_dr_6_range_20-40_msm.png
R_96_dr_6_range_20-40_sm.png
R_96_dr_6_range_-20-20.csv
R_96_dr_6_range_-20-20_Chi_0.99942.txt
R_96_dr_6_range_-20-20_m.png
R_96_dr_6_range_-20-20_msm.png
R_96_dr_6_range_-20-20_sm.png
R_96_dr_6_range_-40--20.csv
R_96_dr_6_range_-40--20_Chi_0.99967.txt
R_96_dr_6_range_-40--20_m.png
R_96_dr_6_range_-40--20_msm.png
R_96_dr_6_range_-40--20_sm.png

6) Open the csv files created by the program. The values within each of the csv file are the raw results from the MatLab script. The file name of the csv document corresponds to the total radius of the program, the bin size(dr), and the range of the axial segment.

7) The csv file will have 3 columns of radial information (histogram count, raw matrix equation values, and smoothed matrix equation values). Copy the third column (smoothed matrix equation values) to a separate excel file.

	A	B	C
1	21	-1.1869	-1.1869
2	23	0.97271	0.75969
3	22.667	2.4933	0.68325
4	18.667	-1.4162	0.19648
5	23	-0.48759	-0.41475
6	27.667	0.65957	1.2208
7	31	3.4905	2.207
8	25.667	2.4708	2.2779
9	22	0.87243	1.5557
10	23.667	1.3237	1.3769
11	25	1.9344	2.2327
12	24.333	3.4399	2.4597
13	16.667	2.0047	2.4583
14	13	1.9302	1.8781
15	8	1.6994	1.3779
16	2	0.50395	0.73446
17	0	0	0

	A	B	C
1	(-40 to -20)	(-20 to 20)	(20 to 40)
2			
3	-1.1869		
4	0.75969		
5	0.68325		
6	0.19648		
7	-0.41475		
8	1.2208		
9	2.207		
10	2.2779		
11	1.5557		
12	1.3769		
13	2.2327		
14	2.4597		
15	2.4583		
16	1.8781		
17	1.3779		
18	0.73446		
19	0		

8) Repeat the last step for the other axial ranges from the other excel files.

	A	B	C
1	(-40 to -20)	(-20 to 20)	(20 to 40)
2			
3	-1.1869	-0.89177	-1.1508
4	0.75969	-0.85905	0.2399
5	0.68325	-0.73929	0.64383
6	0.19648	0.070089	0.74101
7	-0.41475	1.9312	0.4396
8	1.2208	4.8701	1.1513
9	2.207	6.5832	0.64912
10	2.2779	6.3345	0.7658
11	1.5557	3.8766	0.18354
12	1.3769	1.6434	1.106
13	2.2327	0.86393	1.5809
14	2.4597	1.1673	1.8684
15	2.4583	1.6952	1.3469
16	1.8781	1.3457	0.76825
17	1.3779	1.7469	1.6748
18	0.73446	1.1665	1.4493
19	0	0	0

9) These values will have to be normalized to the number of bins in each subsection. For instance, with a bin size of 6nm, the section that ranges from -40nm to -20nm is comprised of 3.33 subregions (bins). Likewise, the section that ranges from -20nm to 20nm is comprised of 6.66 subregions, and the section that ranges from 20nm to 40nm is comprised of 3.33 subregions. Therefore, the values in each of the columns must be divided by 3.33, 6.66, and 3.33 respectively.

(-40 to -20)	(-20 to 20)	(20 to 40)
-0.35607	-0.133766	-0.34524
0.227907	-0.128858	0.07197
0.204975	-0.110894	0.193149
0.058944	0.0105134	0.222303
-0.124425	0.28968	0.13188
0.36624	0.730515	0.34539
0.6621	0.98748	0.194736
0.68337	0.950175	0.22974
0.46671	0.58149	0.055062
0.41307	0.24651	0.3318
0.66981	0.1295895	0.47427
0.73791	0.175095	0.56052
0.73749	0.25428	0.40407
0.56343	0.201855	0.230475
0.41337	0.262035	0.50244
0.220338	0.174975	0.43479
0	0	0

10) Find the maximum value of the whole data set.

(-40 to -20)	(-20 to 20)	(20 to 40)
-0.35607	-0.133766	-0.34524
0.227907	-0.128858	0.07197
0.204975	-0.110894	0.193149
0.058944	0.0105134	0.222303
-0.124425	0.28968	0.13188
0.36624	0.730515	0.34539
0.6621	0.98748	0.194736
0.68337	0.950175	0.22974
0.46671	0.58149	0.055062
0.41307	0.24651	0.3318
0.66981	0.1295895	0.47427
0.73791	0.175095	0.56052
0.73749	0.25428	0.40407
0.56343	0.201855	0.230475
0.41337	0.262035	0.50244
0.220338	0.174975	0.43479
0	0	0
	**Max**	**0.98748**

11) Normalize the values in each column to the maximum value of the data set. For LBR-C, this maximum value is 0.98748.

(-40 to -20)	(-20 to 20)	(20 to 40)
-0.360585	-0.13546	-0.34962
0.2307966	-0.13049	0.072882
0.2075738	-0.1123	0.195598
0.0596913	0.010647	0.225122
-0.126003	0.293353	0.133552
0.3708835	0.739777	0.349769
0.6704946	1	0.197205
0.6920343	0.962222	0.232653
0.4726273	0.588863	0.05576
0.4183072	0.249635	0.336007
0.6783023	0.131233	0.480283
0.7472658	0.177315	0.567627
0.7468404	0.257504	0.409193
0.5705736	0.204414	0.233397
0.418611	0.265357	0.50881
0.2231316	0.177193	0.440303
0	0	0

12) If any negative values remain, remove them as they are noise generated from the axial to radial transformation algorithm. These values are generated due to a lack of particles in the empty regions of the NPC.

(-40 to -20)	(-20 to 20)	(20 to 40)
0	0	0
0.2307966	0	0.072882
0.2075738	0	0.195598
0.0596913	0.010647	0.225122
0	0.293353	0.133552
0.3708835	0.739777	0.349769
0.6704946	1	0.197205
0.6920343	0.962222	0.232653
0.4726273	0.588863	0.05576
0.4183072	0.249635	0.336007
0.6783023	0.131233	0.480283
0.7472658	0.177315	0.567627
0.7468404	0.257504	0.409193
0.5705736	0.204414	0.233397
0.418611	0.265357	0.50881
0.2231316	0.177193	0.440303
0	0	0

13) Then add a radial dimension column "R (nm)". This column will start at half the bin size then increase by the bin size. For LBR-C, with an optimal bin size of 6, the column will start at 3 nm then increase by 6 nm for each density measurement.

R(nm)	(-40 to -20)	(-20 to 20)	(20 to 40)
3	0	0	0
9	0.2307966	0	0.072882
15	0.2075738	0	0.195598
21	0.0596913	0.010647	0.225122
27	0	0.293353	0.133552
33	0.3708835	0.739777	0.349769
39	0.6704946	1	0.197205
45	0.6920343	0.962222	0.232653
51	0.4726273	0.588863	0.05576
57	0.4183072	0.249635	0.336007
63	0.6783023	0.131233	0.480283
69	0.7472658	0.177315	0.567627
75	0.7468404	0.257504	0.409193
81	0.5705736	0.204414	0.233397
87	0.418611	0.265357	0.50881
93	0.2231316	0.177193	0.440303
99	0	0	0

14) To determine the peak density at different radial distances, you will then transfer the data set into Origin (or your graphing program of choice).

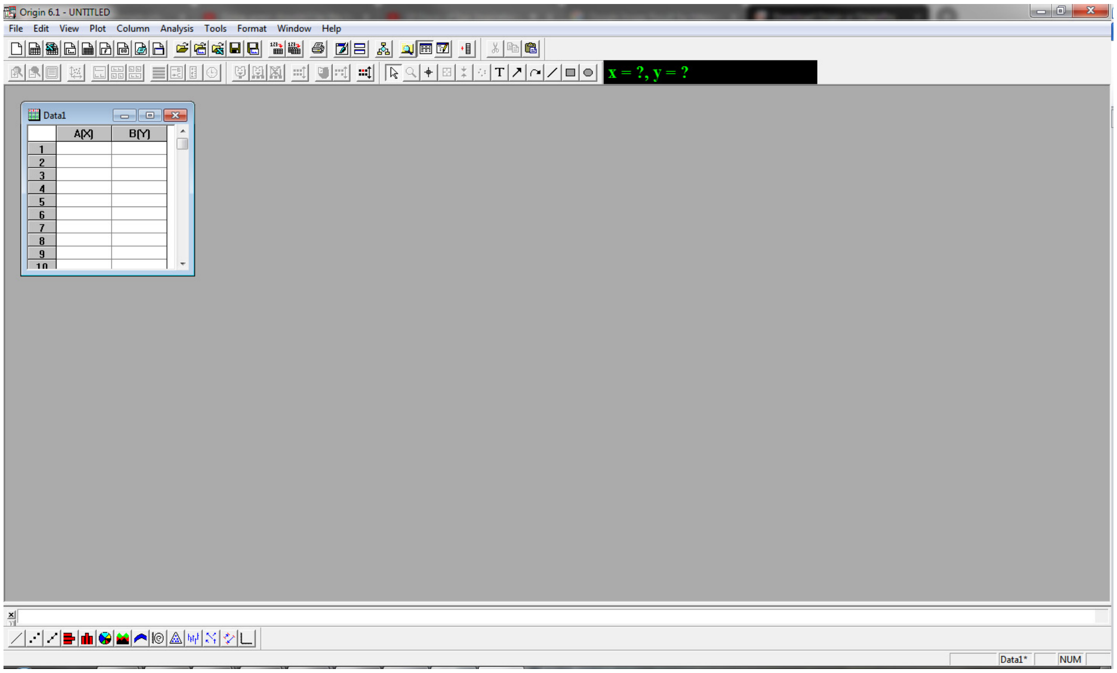

15) Copy the dataset into a new worksheet. With the radial dimension values as (X) and the density values as (Y).

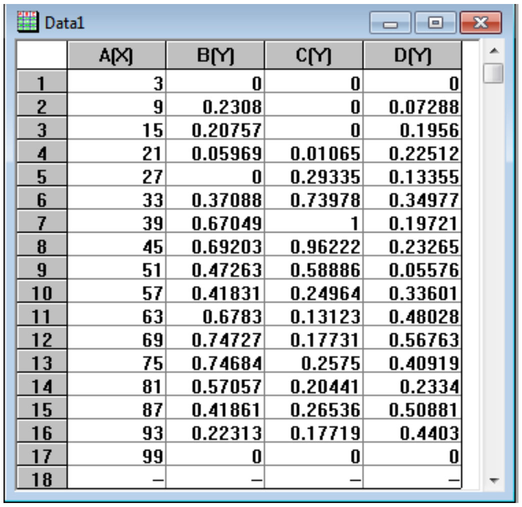
Data1

	A(X)	B(Y)	C(Y)	D(Y)
1	3	0	0	0
2	9	0.2308	0	0.07288
3	15	0.20757	0	0.1956
4	21	0.05969	0.01065	0.22512
5	27	0	0.29335	0.13355
6	33	0.37088	0.73978	0.34977
7	39	0.67049	1	0.19721
8	45	0.69203	0.96222	0.23265
9	51	0.47263	0.58886	0.05576
10	57	0.41831	0.24964	0.33601
11	63	0.6783	0.13123	0.48028
12	69	0.74727	0.17731	0.56763
13	75	0.74684	0.2575	0.40919
14	81	0.57057	0.20441	0.2334
15	87	0.41861	0.26536	0.50881
16	93	0.22313	0.17719	0.4403
17	99	0	0	0
18	–	–	–	–

16) Select the column containing the radial dimension values (For this example, A(X)) and one of the density value columns (For this example, C(Y)).

Data1

	A(X)	B(Y)	C(Y)	D(Y)
1	3	0	0	0
2	9	0.2308	0	0.07288
3	15	0.20757	0	0.1956
4	21	0.05969	0.01065	0.22512
5	27	0	0.29335	0.13355
6	33	0.37088	0.73978	0.34977
7	39	0.67049	1	0.19721
8	45	0.69203	0.96222	0.23265
9	51	0.47263	0.58886	0.05576
10	57	0.41831	0.24964	0.33601
11	63	0.6783	0.13123	0.48028
12	69	0.74727	0.17731	0.56763
13	75	0.74684	0.2575	0.40919
14	81	0.57057	0.20441	0.2334
15	87	0.41861	0.26536	0.50881
16	93	0.22313	0.17719	0.4403
17	99	0	0	0
18	–	–	–	–

17) Then generate a vertical column graph.

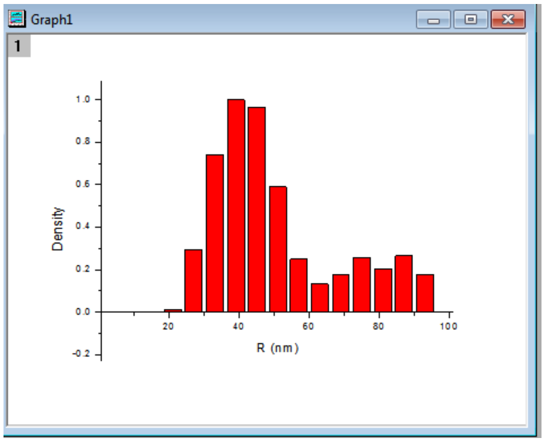

18) Fit the resultant graph to a Gaussian function.

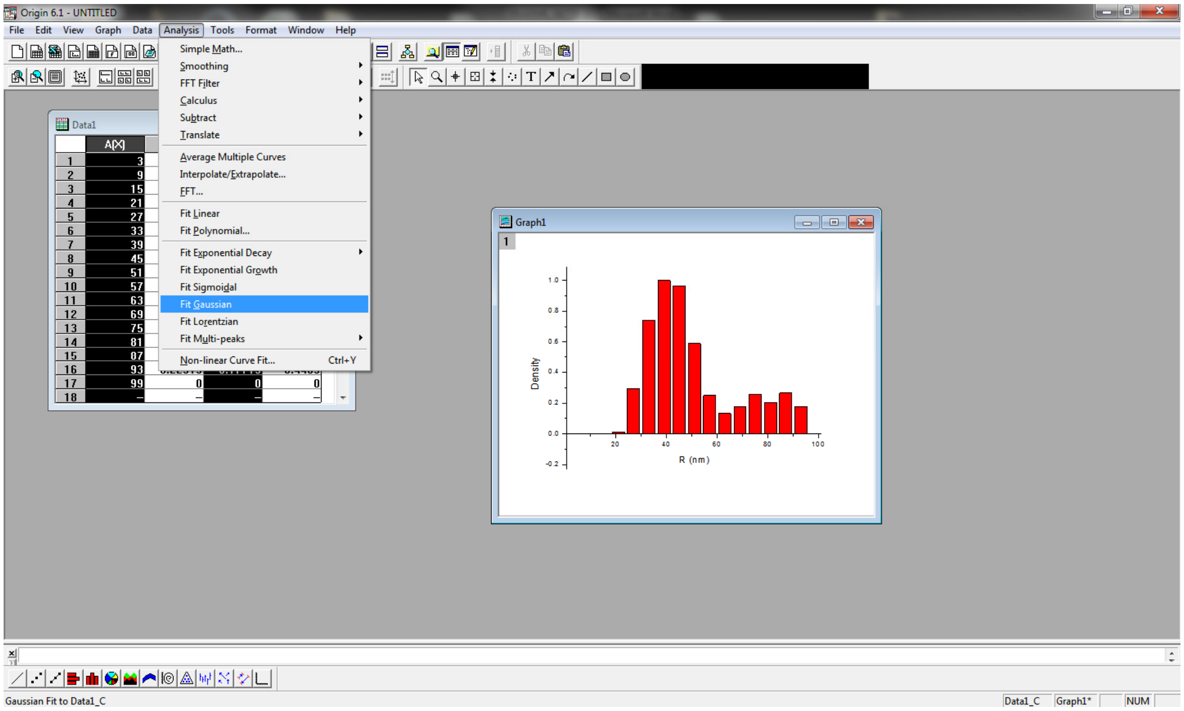

19) The center of the peak will be the mean of the peak density distribution. This value is the transport route with largest density distribution within that axial range. The density distribution of LBR-C within the axial range of -20nm to 20nm will produce a peak mean of 41.25699 ± 0.79089.

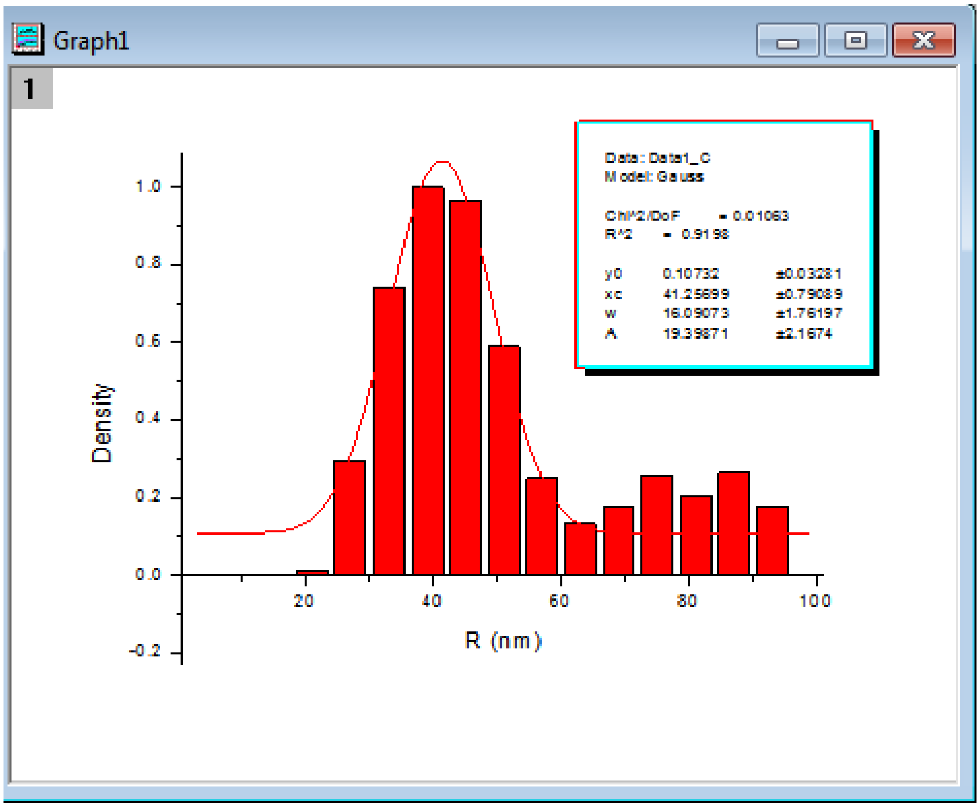

Section V

Simulation to Determine Confidence and Route Precision

1) Similar to section II, Open Spyder – it should have an icon on your desktop or an icon in the program list under the start menu for Windows.

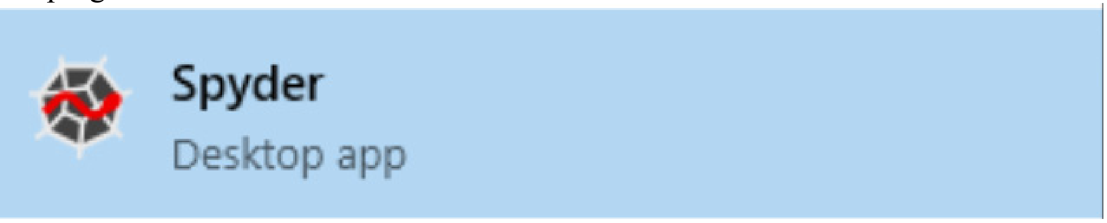

2) Once Spyder starts up, open "simulation_gui.py" using the File>Open menu.

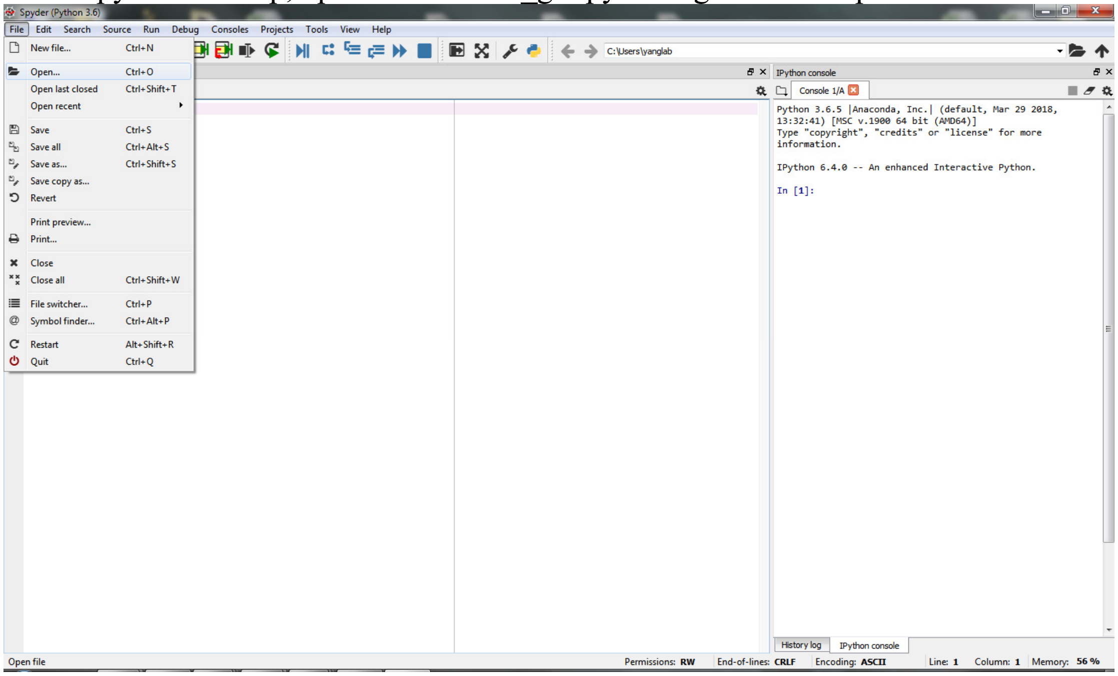

3) Once "simulation_gui.py" is open, click the green "Run file" arrow in the toolbar.

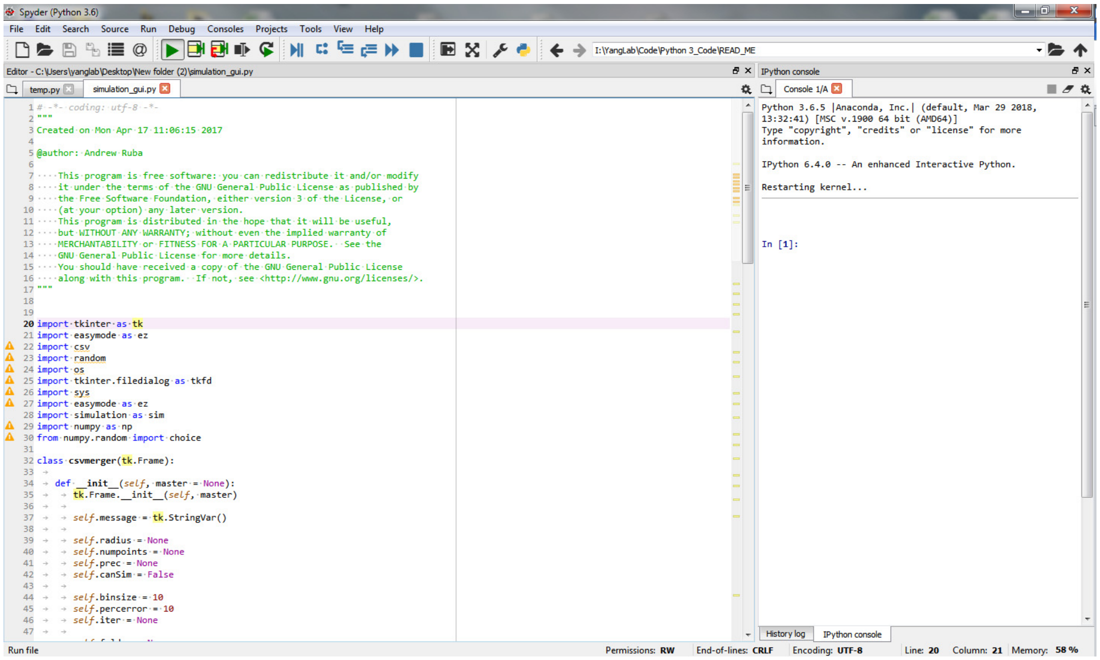

4) When the script runs, a pop-up window will appear.

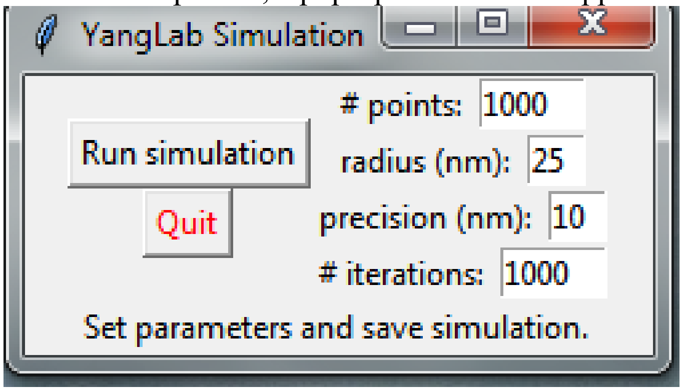

5) Input the following values;
   i. The number of points (# ***points:***).
   ii. The peak radius found from Origin as explained in section III (***radius (nm):***).
   iii. The precision of the your single-molecule localizations (**precision (nm):**).
   iv. The number iterations you would like the simulation to run (# **iterations:**).

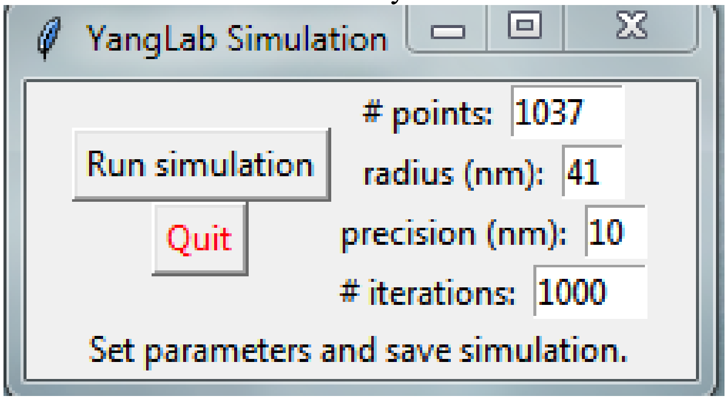

6) Click “Run simulation” after entering the proper integer values for the simulation parameters you would like to run. The optimal bin size will be dynamically calculated according to the parameters and the reproducibility rate and route localization precision will be written into the gui message area.

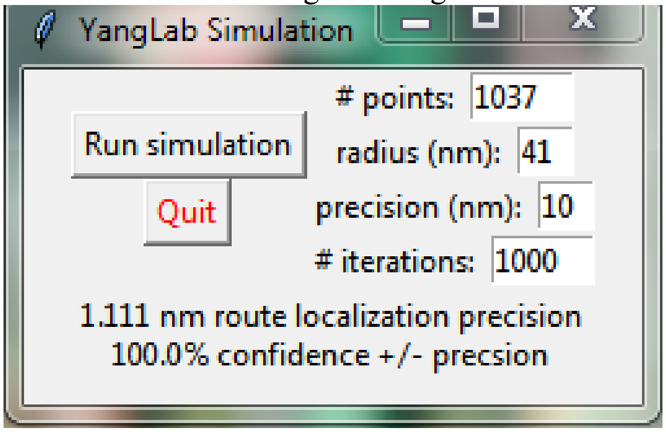

7) Select the “Quit” button to close the pop-up window.
