# Supplementary material for: Nucleoplasmic signals promote directed transmembrane protein import simultaneously via multiple channels of nuclear pores
Source: Nat Commun. 2020 May 4;11:2184. doi: 10.1038/s41467-020-16033-x (PMC7198523; doi:10.1038/s41467-020-16033-x)
Supplement: Supplementary file 3 — Reporting Summary [file 41467_2020_16033_MOESM3_ESM.pdf]

nature research

Corresponding author(s): Weidong Yang
Last updated by author(s): 04/07/2020

Reporting Summary

Nature Research wishes to improve the reproducibility of the work that we publish. This form provides structure for consistency and transparency in reporting. For further information on Nature Research policies, see Authors & Referees and the Editorial Policy Checklist.

Statistics

For all statistical analyses, confirm that the following items are present in the figure legend, table legend, main text, or Methods section.

n/a	Confirmed	
☐	☒	The exact sample size (*n*) for each experimental group/condition, given as a discrete number and unit of measurement
☐	☒	A statement on whether measurements were taken from distinct samples or whether the same sample was measured repeatedly
☐	☒	The statistical test(s) used AND whether they are one- or two-sided<br>*Only common tests should be described solely by name; describe more complex techniques in the Methods section.*
☐	☒	A description of all covariates tested
☐	☒	A description of any assumptions or corrections, such as tests of normality and adjustment for multiple comparisons
☐	☒	A full description of the statistical parameters including central tendency (e.g. means) or other basic estimates (e.g. regression coefficient) AND variation (e.g. standard deviation) or associated estimates of uncertainty (e.g. confidence intervals)
☐	☒	For null hypothesis testing, the test statistic (e.g. *F*, *t*, *r*) with confidence intervals, effect sizes, degrees of freedom and *P* value noted<br>*Give P values as exact values whenever suitable.*
☐	☒	For Bayesian analysis, information on the choice of priors and Markov chain Monte Carlo settings
☒	☐	For hierarchical and complex designs, identification of the appropriate level for tests and full reporting of outcomes
☒	☐	Estimates of effect sizes (e.g. Cohen's *d*, Pearson's *r*), indicating how they were calculated

Our web collection on statistics for biologists contains articles on many of the points above.

Software and code

Policy information about availability of computer code

Data collection	Simulation code can be found at: https://github.com/andrewruba/YangLab.
Data analysis	The Slidebook software package was purchased from Intelligent Imaging Innovations. The GLIMPSE software package is a gift from the Gelles Lab (Brandies University). The ImageJ plugin ThunderSTORM was downloaded from an open source zitmen.github.io/thunderstorm/). Simulation source code has been provided at: https://github.com/andrewruba/YangLab.

For manuscripts utilizing custom algorithms or software that are central to the research but not yet described in published literature, software must be made available to editors/reviewers. We strongly encourage code deposition in a community repository (e.g. GitHub). See the Nature Research guidelines for submitting code & software for further information.

Data

Policy information about availability of data

All manuscripts must include a data availability statement. This statement should provide the following information, where applicable:

- Accession codes, unique identifiers, or web links for publicly available datasets
- A list of figures that have associated raw data
- A description of any restrictions on data availability

All raw data for figures and tables in the manuscript or the supplementary materials is available upon requests. Simulation source code has been provided at: https://github.com/andrewruba/YangLab.

Field-specific reporting

Please select the one below that is the best fit for your research. If you are not sure, read the appropriate sections before making your selection.

☒ Life sciences ☐ Behavioural & social sciences ☐ Ecological, evolutionary & environmental sciences

For a reference copy of the document with all sections, see nature.com/documents/nr-reporting-summary-flat.pdf

Life sciences study design

All studies must disclose on these points even when the disclosure is negative.

Sample size

At least 1,000 single-molecule locations for transiting molecules were collected from up to 10 different cells. The sufficiency for the sample size has been verified by Monte Carlo simulations.

Data exclusions

Single-molecule data with localization precision of <10 nm have been used to determine the 3D nuclear transport routes for INM proteins due to the following rationale:
To ensure a high reproducibility of 3D spatial probability density maps obtained for each membrane protein candidate, extensive measurements were conducted by combining experimental data and computational simulation. It is important to note that route localization precisions are different from single-molecule localization precision. In detail, the route localization precision is determined by two parameters: one is the number of single-molecule locations and the other is single-molecule localization precision. As shown in Supplementary Fig. 10, simulated data was used to estimate the minimum number of single-molecule localizations required to generate a reliable 3D probability density map for routes of 25 nm (central channel transport) or 40 nm (peripheral channel transport) radial distances. A single-molecule localization precision of <10 nm was used to reflect the precision of our experimentally collected data. We used three different sample sizes (100, 200, and 500 points) and converted the 2D data to 3D by using our transformation algorithm. Peak positions were fitted for data generated from each of the three sample sizes. 100 fits were used to determine the peak position and the standard deviation is used for the route localization precision. Simulation code can be found at: https://github.com/andrewruba/YangLab.

Replication

To ensure a high reproducibility of 3D spatial probability density maps obtained for each membrane protein candidate, extensive measurements were conducted by combining experimental data and computational simulation.

Randomization

Single-molecule locations were selected randomly and purely based on their localization precision.

Blinding

Yes, we were blinded to group allocation during data collection.

Reporting for specific materials, systems and methods

We require information from authors about some types of materials, experimental systems and methods used in many studies. Here, indicate whether each material, system or method listed is relevant to your study. If you are not sure if a list item applies to your research, read the appropriate section before selecting a response.

Materials & experimental systems

n/a	Involved in the study
☐	☒ Antibodies
☐	☒ Eukaryotic cell lines
☒	☐ Palaeontology
☒	☐ Animals and other organisms
☒	☐ Human research participants
☒	☐ Clinical data

Methods

n/a	Involved in the study
☒	☐ ChIP-seq
☒	☐ Flow cytometry
☒	☐ MRI-based neuroimaging

Antibodies

Antibodies used

Cells were injected with either WGA (ThermoFisher, W32466, at 1.33 μM) or anti-gp210 (Novus Biologicals, NB100-93336, at 1.33 μM) and incubated for 30 min at 37°C in transport buffer before imaging. Rabbit primary antibody anti-GFP (A-11122) and mouse anti-γ-tubulin (MA1-850) were purchased from Thermo Fisher. For Western blotting IR680 and IR800 conjugated goat anti-rabbit antibodies (LI-COR Biosciences) were used.

Validation

Purified bacterial N-terminal fusion protein and Specificity Recognizes rat & xenopus gp210. This product is for research use only and is not approved for use in humans or in clinical diagnosis. Primary Antibodies are guaranteed for 1 year from date of receipt.

For rabbit primary antibody anti-GFP (A-11122), a primary antibody from Thermo Fisher is an immunoglobulin that specifically binds to a particular protein or other biomolecule of research interest for the purpose of purifying or detecting and measuring it. Primary antibodies are developed as polyclonal or monoclonal antibodies using mouse, rat, rabbit, goat, and other animal species as hosts. They are produced and supplied in various forms, ranging from crude antiserum to antigen-purified preparations. Primary antibodies for frequently researched targets are also available conjugated to fluorescent dyes or biotin.

For mouse anti-γ-tubulin (MA1-850), MA1-850 detects gamma Tubulin from human, mouse, monkey, and rat samples. MA1-850 has been successfully used in Western blot, immunoprecipitation, and immunofluorescence procedures. The MA1-850 immunogen is a recombinant protein containing residues A A T R P D Y I S W G T Q E Q of human gamma Tubulin.

For IR680 and IR800, isolation of specific antibodies was accomplished by affinity chromatography using pooled rabbit IgG covalently linked to agarose. Based on ELISA and flow cytometry, this antibody reacts with the heavy and light chains of rabbit IgG, and with the light chains of rabbit IgM and IgA. This antibody was tested by dot blot and and/or solid-phase adsorbed for minimal cross-reactivity with human, mouse, rat, sheep, and chicken serum proteins, but may cross-react with immunoglobulins from other species. The conjugate has been specifically tested and qualified for Western blot and In-Cell Western™ assay applications.

Eukaryotic cell lines

Policy information about cell lines

Cell line source(s)	Wild-type HeLa (ATCC) and stably transfected HeLa cell lines containing mCherry tagged PoM121 proteins were grown in DMEM, high glucose, GlutaMAX Supplement (Life Technologies), 10% fetal bovine serum (Fischer Scientific), 1% penicillin-streptomycin (Thermo Fischer). Cells were transfected by electroporation (Bio-Rad GenePulser Xcell) following the manufacturer's protocol.
Authentication	Obtaining and using low-passage cell lines from ATCC after following the standard authentication procedure, which includes the following tests: 1) Morphology check by microscope; 2) Growth curve analysis; 3) Species verification by Isoenzymology; 4) Identity verification with STR analysis (DNA fingerprinting) for human cell lines; and 5) Mycoplasma detection
Mycoplasma contamination	All cell lines tested negative for mycoplasma contamination.
Commonly misidentified lines (See ICLAC register)	No commonly misidentified lines used in this study.
