# Supplementary material for: Nucleoplasmic signals promote directed transmembrane protein import simultaneously via multiple channels of nuclear pores
Source: Nat Commun. 2020 May 4;11:2184. doi: 10.1038/s41467-020-16033-x (PMC7198523; doi:10.1038/s41467-020-16033-x)
Supplement: Supplementary file 4 — Description of Additional Supplementary Files [file 41467_2020_16033_MOESM4_ESM.pdf]

Description of Additional Supplementary Files

Supplementary Movie 1. 3D rotating movie demonstrating the three distinct pathways of various molecules through the NPC (white mesh figure). The blue density cloud represents the pathway taken by small soluble molecules as they passively diffuse through the axial center of the NPC. The green density cloud represents the transport route of transport receptors and NLS containing soluble proteins along the edge of the central channel of the NPC. The red-yellow density cloud represents the transport route of INM transmembrane proteins that do not contain an NLS or ID region.

Supplementary Movie 2. Dual-channel co-tracking of GFP-LBR-RFP by SPEED microcopy. LBR was tagged with EGFP and RFP on the N- and C-terminal ends respectively and tracked using SPEED microscopy. The first 16-ms movie is a representative single-molecule raw video of the EGFP (left panel) and RFP (right panel) as they move through NPC (solid yellow dot). The second 16-ms movie shows the EGFP (green, left panel), RFP (red, middle panel), and the merged locations of both EGFP and RFP (right panel) as they transit through the NPC (solid white dot).

Supplementary Movie 3. 3D rotating movie demonstrating the pathway the N- and C-terminus of LBR obtained by co-tracking experiments of GFP-LBR-RFP. Both the EGFP tagged N-terminal and RFP tagged C-terminal routes overlaid ($\pm$5 nm error) over the NPC (white mesh) indicating that LBR simultaneously uses two different channels as it transits through the NPC. The pink-blue density cloud represents the transport route of the EGFP-tagged N-terminus of LBR as it moves through the central channel of the NPC. The red-yellow density cloud represents the transport route of the RFP-tagged C-terminus of LBR as it moves through the peripheral channel of the NPC.
